# Single‐neuron analysis of aging‐associated changes in learning reveals impairments in transcriptional plasticity

**DOI:** 10.1111/acel.14228

**Published:** 2024-06-24

**Authors:** Kerriann K. Badal, Abhishek Sadhu, Bindu L. Raveendra, Carrie McCracken, Sebastian Lozano‐Villada, Amol C. Shetty, Phillip Gillette, Yibo Zhao, Dustin Stommes, Lynne A. Fieber, Michael C. Schmale, Anup Mahurkar, Robert D. Hawkins, Sathyanarayanan V. Puthanveettil

**Affiliations:** ^1^ Department of Neuroscience The Herbert Wertheim UF Scripps Institute for Biomedical Innovation & Technology Jupiter Florida USA; ^2^ Integrated Biology Graduate Program Florida Atlantic University Jupiter Florida USA; ^3^ The Institute for Genome Sciences University of Maryland School of Medicine Baltimore Maryland USA; ^4^ Harriet L. Wilkes Honors College Florida Atlantic University Jupiter Florida USA; ^5^ National Resource for Aplysia University of Miami Rosenstiel School of Marine, Atmospheric, and Earth Sciences Miami Florida USA; ^6^ Department of Neuroscience Columbia University New York New York USA; ^7^ New York State Psychiatric Institute New York New York USA; ^8^ Present address: Center for Alzheimer's and Neurodegenerative Diseases, Peter O’Donnell Jr. Brain Institute University of Texas Southwestern Medical Center Dallas Texas USA

**Keywords:** gene expression, molecular biology of aging, neuroscience, senescence

## Abstract

The molecular mechanisms underlying age‐related declines in learning and long‐term memory are still not fully understood. To address this gap, our study focused on investigating the transcriptional landscape of a singularly identified motor neuron L7 in Aplysia, which is pivotal in a specific type of nonassociative learning known as sensitization of the siphon‐withdraw reflex. Employing total RNAseq analysis on a single isolated L7 motor neuron after short‐term or long‐term sensitization (LTS) training of Aplysia at 8, 10, and 12 months (representing mature, late mature, and senescent stages), we uncovered aberrant changes in transcriptional plasticity during the aging process. Our findings specifically highlight changes in the expression of messenger RNAs (mRNAs) that encode transcription factors, translation regulators, RNA methylation participants, and contributors to cytoskeletal rearrangements during learning and long noncoding RNAs (lncRNAs). Furthermore, our comparative gene expression analysis identified distinct transcriptional alterations in two other neurons, namely the motor neuron L11 and the giant cholinergic neuron R2, whose roles in LTS are not yet fully elucidated. Taken together, our analyses underscore cell type‐specific impairments in the expression of key components related to learning and memory within the transcriptome as organisms age, shedding light on the complex molecular mechanisms driving cognitive decline during aging.

## Graphical abstract

The image illustrates a single‐neuron total RNA sequencing approach designed to uncover molecular insights into age‐related learning impairments. We studied short‐term and long‐term sensitization, a type of nonassociative learning, in the sea slug *Aplysia californica* at three distinct ages. Our molecular analyses identified mRNAs and lncRNAs that were differentially expressed during sensitization. These results indicate that transcriptional plasticity associated with learning is compromised with aging.

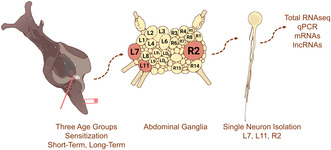

## 1 INTRODUCTION

Advancements in medicine have significantly extended human life expectancy, but they have also introduced new health challenges, particularly age‐related cognitive decline, and the emergence of debilitating diseases. As modern medicine endeavors to confront these challenges, a critical focus emerges on unraveling the molecular underpinnings of the aging process and exploring avenues for its potential reversal, with the prospect of substantially enhancing our quality of life.

Decades of research, utilizing diverse animal models including *Caenorhabditis elegans* (Chen et al., 2013; Wirak et al., 2022), Drosophila (Pacifico et al., 2018; Tonoki & Davis, 2015), Aplysia (Greer et al., 2018; Kadakkuzha et al., 2013; Moroz & Kohn, 2010), rodents (Mota et al., 2019; Ximerakis et al., 2019), and humans (Adewale et al., 2021; Cox et al., 2021), have unveiled numerous molecular and cellular changes underlying aging in the nervous system. These changes encompass alterations in transcription, translation, the epigenome, and synaptic function and plasticity (Azam et al., 2021; Foster, 2002; Mattson & Arumugam, 2018; Rizzo et al., 2014; Schimanski & Barnes, 2010). Of particular significance are transcriptional changes, as they form the basis for subsequent modifications in cellular signaling and intercellular communication. Additionally, the activation of gene expression changes in specific neurons plays a central role in learning and long‐term memory storage (LTM) (Kandel, 2001). This dynamic alteration in the transcriptional state, termed “transcriptional plasticity,” is essential for the establishment of learning and LTM, serving as an adaptive response to environmental stimuli (Brennan et al., 2022; Stern et al., 2007).

Despite comprehensive descriptions of large‐scale transcriptional and epigenetic changes associated with aging in the nervous system, there is limited understanding of the aging‐related changes occurring in individual neurons within a neural circuit during learning (Aging Atlas Consortium, 2021; Allen et al., 2023; Li et al., 2019; Ximerakis et al., 2019). Existing gene expression datasets on neuronal aging lack specificity regarding circuit‐specific or neuron‐specific changes relevant to learning and memory storage. To bridge this knowledge gap, we capitalized on the advantages offered by identified neurons involved in learning within the sea slug *Aplysia californica*. This marine organism serves as a neurobiological model, shedding light on the cellular and molecular mechanisms of learning and LTM (Barco et al., 2006; Baxter & Byrne, 2006; Byrne & Hawkins, 2015; Glanzman, 2008; Kandel, 2001; Lee et al., 2008; Lyons, 2011; Michel & Lyons, 2014; Reissner et al., 2006; Sossin, 2008). Importantly, the behavioral learning of the siphon withdrawal reflex (SWR) in Aplysia is well‐characterized (Antonov et al., 1999; Carew et al., 1981; Frost et al., 1985). The SWR, a defensive reflex, undergoes both nonassociative learning (sensitization) and associative learning (conditioning) (Bailey & Chen, 1989; Carew et al., 1971, 1981; Frost et al., 1985; Pinsker et al., 1973; Scholz & Byrne, 1987). During sensitization siphon withdrawals triggered by weak stimuli are augmented by training with a stronger stimulus such as tail shock. A single shock results in short‐term sensitization (STS), lasting several minutes, while four spaced shocks produce long‐term sensitization (LTS), lasting several days.

To unravel the molecular mechanisms underlying age‐related changes in transcriptional modulation crucial for learning, we focused our investigation on sensitization of SWR. Specifically, we aimed to assess the modifications in mRNA and long‐noncoding RNA (lncRNA) expression within the L7 motor neuron (L7MN), a key component of the SWR circuitry. Employing RNAseq analyses, we meticulously examined the transcriptional landscape of L7MN following STS and LTS across three different age groups. We began with identifying the elements of transcriptional plasticity in L7MN of 8‐month‐old Aplysia, subsequently comparing these findings with L7MN isolated from animals aged 10 and 12 months. In line with established literature on age‐related behavioral changes in Aplysia (Bailey et al., 1983; Kempsell & Fieber, 2015a, 2015b; Rattan & Peretz, 1981), we pinpointed specific deficiencies in LTS during aging. Our gene expression analyses yielded insights into impairments in both coding and long‐noncoding transcriptome of L7MN. Moreover, we uncovered shared and neuron‐specific alterations in gene expression related to learning and aging in two additional neurons: motor neuron L11 and cholinergic neuron R2. Despite their roles in STS or LTS remaining elusive, these findings contribute to our understanding of the complex interplay between learning, aging, and transcriptional regulation within the Aplysia nervous system.

## 2 RESULTS

### 2.1 Behavioral training showed impairments in LTS of the SWR

**FIGURE 1:**
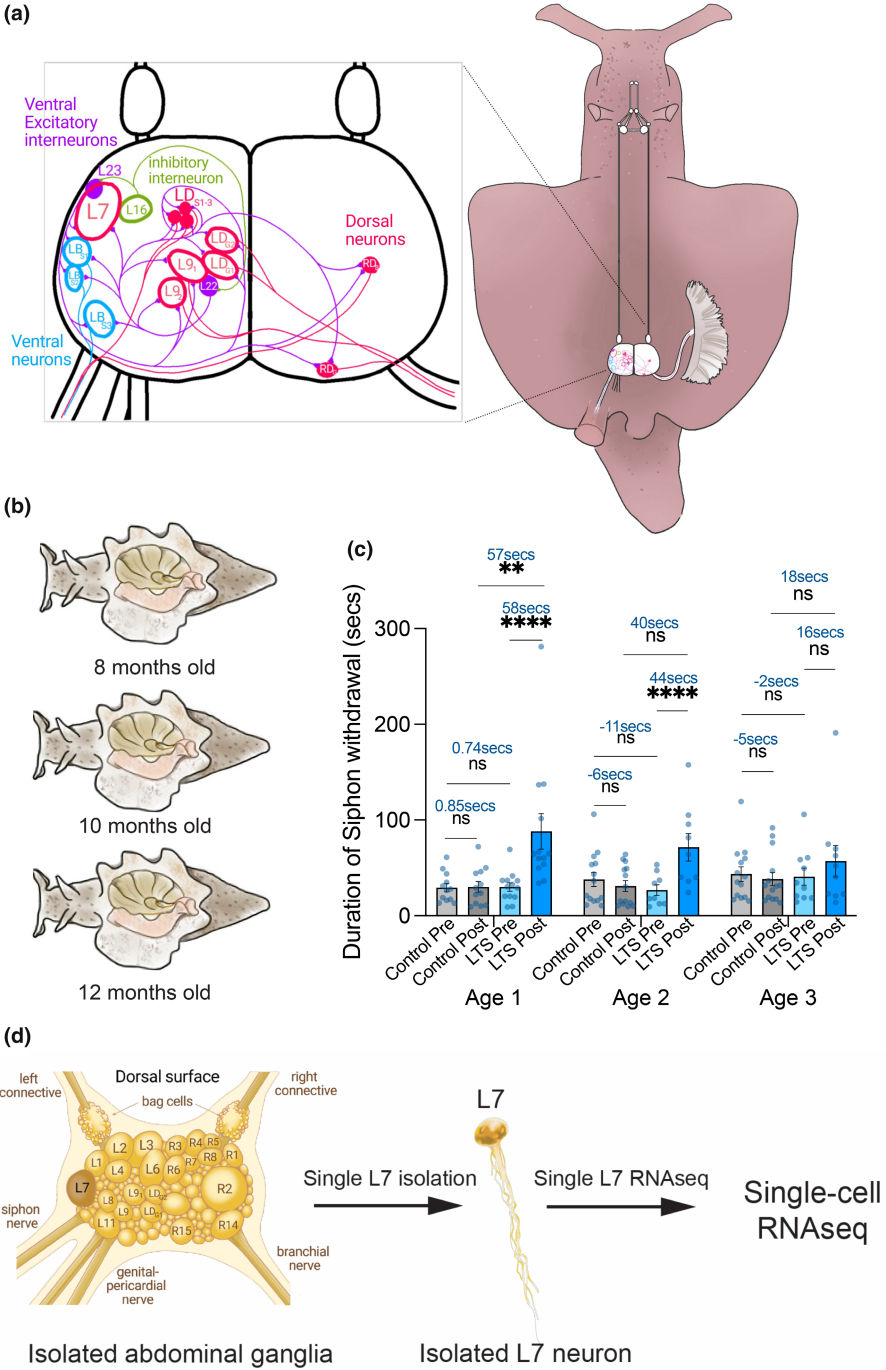
Overview of single‐neuron analysis of aging‐associated changes in learning. (a) Schematic of the known components of the gill siphon withdrawal reflex in Aplysia abdominal ganglia adopted from Kupfermann et al. (1974). (b) Age groups 8 months (Age 1), 10 months (Age 2), 12 months (Age 3). (c) Bar graphs showing the average duration of siphon withdrawal from the stimulus to the time the siphon begins to relax before (Pre) and 24 h after (Test) long‐term sensitization training (LTS) or no shock control in three age groups. The number of animals used for analysis is shown in the bar graphs. Data was first log transformed and then a three‐way ANOVA was performed followed by individual post hoc comparisons. There was a significant Pre versus Post × shock versus control × age group three‐way interaction overall and multiple significant individual comparisons indicated in the figure (**p < 0.001, ****p < 0.0001, NS: nonsignificant. Error bars are SEM, see Table S1). (d) Schematic representation of the workflow for single L7M isolation to RNAseq from trained (short‐term and long‐term sensitization) and untrained animals age groups 1–3. See also related Figure S1 and Table S1.

Since LTM formation requires gene expression and new protein synthesis (Byrne & Hawkins, 2015; Emptage & Carew, 1993; Ghirardi et al., 1995; Kandel, 2001; Sutton & Carew, 2000; Sutton & Schuman, 2006), we searched for transcriptomic alterations that are associated with aging‐related changes in LTS. To unravel the transcriptomic underpinnings of aging‐related deficits in LTS, we conducted total RNAseq analysis on L7M extracted from trained (STS or LTS) animals and untrained age‐matched controls spanning different age groups. Figure 1a outlines the known components of the gill‐SWR. Importantly, the monosynaptic connection between the siphon sensory neurons and motor neuron L7 plays a pivotal role in the learning and LTM of the SWR (Bailey & Chen, 1989; Frost et al., 1985, 1997). Remarkably, within the animal, there exists only one L7MN, identifiable by its distinct size and location in the abdominal ganglion.

We established two cohorts of animals evaluating changes in the SWR and gene expression at 8 months (age group 1, sexually mature adults), 10 months (age group 2, late mature), and 12 months (age group 3, senescent). Aplysia typically live up to 12–14 months under normal conditions at the National Aplysia Resource Facility, with potential lifespan extension through dietary and temperature adjustments (Stommes et al., 2005). Initially, we examined LTS of the SWR, using no‐shock animals from the same cohorts for comparison. Briefly, siphon‐withdrawal duration was assessed before and 24 h after LTS training or control in age groups 1, 2, and 3. A three‐way ANOVA analysis of pre versus posttest siphon withdrawal duration following LTS training in age groups 1, 2, and 3 revealed a significant difference in age groups 1 and 2, indicating the retention of LTS (Figure 1b,c; Table S1). Conversely, age group 3 animals failed to demonstrate LTS retention, with a significant 3‐way interaction suggesting age‐related impairments in learning and LTM (Figure 1c; Table S1).

To ascertain whether STS is also influenced by aging, we established another cohort of animals and compared SWR duration in age groups 1 and 3 after STS training. Our findings unveiled that age group 3 Aplysia did not retain STS, as evidenced by the lack of a significant difference in pre‐ versus posttest siphon withdrawal duration and a significant 3‐way interaction (Figure S1; Table S1). Collectively, these observations suggest that aging adversely affects both short and long‐term memory in Aplysia.

### 2.2 Short‐term and LTS training induces specific changes in long‐noncoding and coding transcriptomes

As mentioned earlier, the formation of LTM necessitates specific alterations in the transcriptome. To illuminate the transcriptomic basis of LTS and STS, we conducted total RNAseq analysis of the transcriptome of individual L7MN isolated from trained and untreated animals one hour after training (Figure 1d). This analysis aimed to uncover differentially modulated messenger RNAs (mRNAs) and long noncoding RNAs (lncRNAs). Given the known critical roles of lncRNAs in epigenomic regulation and transcriptional control, identifying lncRNAs modulated by learning and aging in a single identified key neuron mediating learning process promises to offer fresh insights into the impact of aging and learning on both coding and noncoding transcriptome dynamics.

**FIGURE 2:**
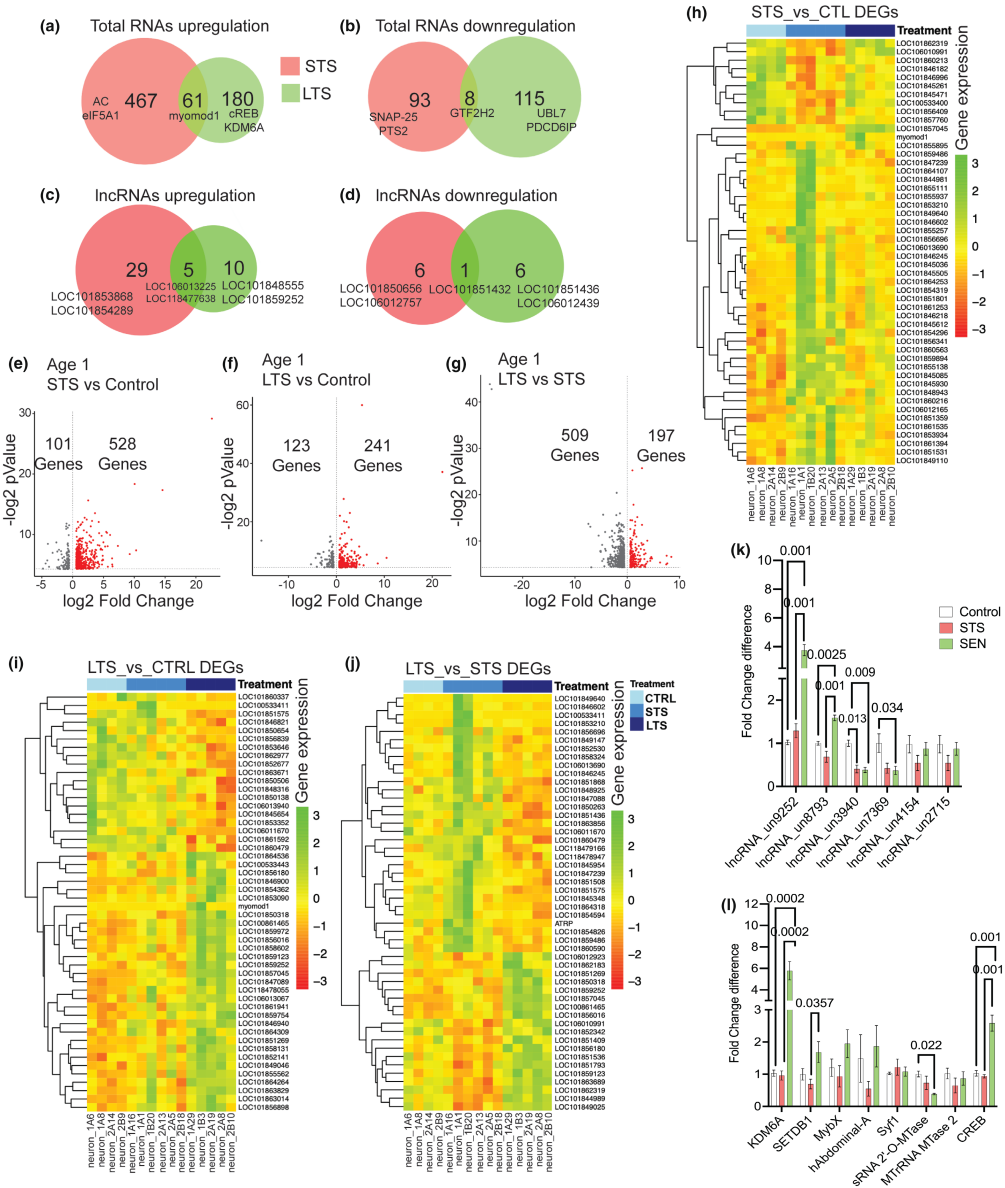
RNAseq analysis of L7MN reveals specific changes in the expression of mRNAs and long‐noncoding RNAs (lncRNAs) following STS and LTS training. Venn diagrams showing (the numbers indicate unique and common differentially expressed genes (DEGs) in 8‐month‐old animals. (a) Upregulated, (b) downregulated in response to short‐term sensitization, STS and long‐term sensitization, LTS (*p* <0.05; the numbers indicate unique and common DEGs) (see Figures S2–S6, Table S2). Venn diagrams showing differentially expressed lncRNAs (c) upregulated, (d) downregulated in response to STS and LTS (*p* <0.05). Differentially expressed genes are ranked in a volcano plot according to their statistical‐log2 *p* (*y‐*axis) and their relative abundance ratio (log2 fold change) between up‐ and downregulated (*x‐*axis). Red dots indicate significantly regulated genes (false discovery rate, <0.01; s0 = 1; *p* <0.05) (see Table S2). Volcano plots of (e). Control versus STS DEGs, (f) Control versus LTS DEGs, (g). LTS versus STS DEGs (see Table S2). Heatmaps showing the normalized and scaled expression values of the top 50 differentially expressed genes when ranked by *p*‐value. The color gradient from green to red represents high to low expression levels across the samples. The genes are ordered by hierarchical clustering using Euclidean distance and complete clustering method while the samples are ordered by condition, (h) STS versus Control, (i) LTS versus Control, (j) LTS versus STS (see Table S2). qPCR validation of selected candidates from RNAseq data, (k) lncRNAs, (l) mRNAs. Relative gene expression levels are exhibited as the mean fold change, with error bars showing the SEM. One‐way ANOVA followed by Tukey's post hoc test. *N* = 4, *p* values are shown in the bar graphs (see Table S2).

Figures S2–S6 provide alignment and mapping statistics of L7MN RNAseq data. The analysis of the L7MN transcriptome at age 1 revealed 1314 unique RNA sequences, including 82 lncRNAs, from L7MNs isolated from STS‐ and LTS‐trained animals (*p* < 0.05, >1.5‐fold up‐ or downregulated, Table S2). Bioinformatics analysis identified 629, 364, and 706 differentially expressed genes (DEGs) between STS versus control, LTS versus control, and LTS versus STS, respectively, covering 2.15%, 1.24%, and 2.41% of all annotated genes (AplCal3.0; GCF_000002075.1; total 29,270 transcripts) (Figure 2a–j; Table S2). Venn diagrams depicted in Figure 2 suggest that STS and LTS training paradigms alter the transcription of specific populations of mRNAs and lncRNAs.

Briefly, we find that the DEGs we identified are involved in strengthening of the existing synaptic connections. For example, STS training has led to upregulation of RNA transcripts, among which ~6.2% are lncRNAs, among mRNAs ~5% are related to synapse function, ~3.2% are related to transcription/translation, ~6% are kinases or phosphatases. Examples of STS specific regulated mRNAs include multi drug resistance‐associated protein 1 (LOC101849640), eukaryotic translation initiation factor 5A (LOC101854486) and other translation initiation factors (LOC101854486, LOC101853210, LOC101850385), adenylate cyclase (LOC101859667, LOC101860364), adhesion G protein‐coupled receptor L1 (LOC101862738), several cytochrome P450s (LOC101853171, LOC101851093, LOC101852221, LOC101861801, LOC101864543), DNA polymerase alpha catalytic subunit isoform (LOC101861408), FMRFamide receptor (LOC101852874, LOC101859894), and syntaxin‐7 (LOC101861711). Similarly we identified upregulation of 34 lncRNAs of which 5 are commonly upregulated in LTS and STS (Table S2). By contrast LTS training resulted in the upregulation of 241 transcripts relative to long‐term memory formation/ consolidation, ~5.5% are lncRNA, ~10.5% are synaptic proteins, ~8.8% are involved in transcription or translation and ~ 4.4% are kinase or phosphatases. cAMP‐responsive element‐binding protein (CREB, LOC100861465), CREB3 regulatory factor isoform (LOC101858375), probable G‐protein coupled receptor 83 (LOC101855582), calcium/calmodulin‐dependent protein kinase type 1B (LOC101845492), G‐protein coupled receptor GRL101 (LOC101858538), glutamate receptor 2 (LOC100533395), calcium/calmodulin‐dependent protein kinase type 1B (LOC101845492), acetylcholine receptor subunit alpha‐type acr‐16‐like precursor (LOC106012547), lysine‐specific demethylase 6A (LOC101851269) were found upregulated upon LTS, along with a total of 15 lncRNAs (Table S2). Moreover, when comparing LTS to STS, we found long‐term memory related DEGs such as sonic hedgehog protein A (LOC101856180), which has been shown to be activated in the rodent amygdala during learning (Hung et al., 2014), vacuolar protein sorting‐associated protein isoform (LOC101859486), which encodes for proteins related to MIT, a domain contained within Microtubule Interacting and Trafficking molecules (NIH Gene database) and cAMP responsive element‐binding protein (CREB1) (LOC100861465).

We identified several commonly upregulated DEGs during STS and LTS example: muscle contracting myomod1 (myomodulin neuropeptides 1 precursor), a response associated with escape behavior due to sensitization (Stopfer & Carew, 1988) as well as differentially modulated genes (Table S2). For example, different isoforms of FMRFamide receptor, mucin‐5 AC, pedal peptide 2, snRNA_U4 spliceosomal RNA, upregulated during STS, were found to be downregulated during LTS. Similarly, isoforms of calcyphosin‐like protein, multiple epidermal growth factor‐like domains protein 10 downregulated upon STS were found upregulated in LTS. Several lncRNAs were also found to be downregulated in STS and LTS. Examples of downregulated transcripts in LTS include dual specificity protein phosphatase 14 (LOC101848709), syntenin‐1 (LOC101854063), small RNA 2‐O‐methyltransferase (LOC101850673). Taken together, these results show that STS and LTS alter expression of specific sets of mRNAs and lncRNAs in L7MN. Several known genes involved in memory processes (examples: CREB, CaMK II, lysine demethylase) are upregulated in L7MN following LTS unlike STS. While the role of lncRNAs in these STS and LTS are not known, our analysis suggests that lncRNAs are targets of transcriptional modulation during learning and LTM in *Aplysia*.

### 2.3 Validation of differential regulation of lncRNAs and mRNAs by STS and LTS

We next validated our RNAseq data in L7MN isolated following another round of STS and LTS training. For the detailed analyses and follow up validations of mRNAs, we focused on transcripts known to have a role in transcription, and RNA processing. Based on the fold enrichments and known functions in learning and LTM relevant process, we selected eight mRNAs (five upregulated and three downregulated) based on their role in transcription (lysine‐specific demethylase 6A [KDM6A]), histone‐lysine N‐methyltransferase SETDB1 (SETDB1), myb‐like protein X (MybX), homeobox protein abdominal‐A homolog (hAbdominal‐A), cAMP responsive element‐binding protein (CREB) and RNA processing (pre‐mRNA‐splicing factor syf1 homolog (Syf1), small RNA 2′‐O‐methyltransferase sRNA (2'‐O‐MTase) and mitochondrial rRNA methyltransferase 2 [Mt rRNA MTase2]), and six lncRNAs (two upregulated and four downregulated) and analyzed the gene expression levels by qPCR analysis of single L7MN. All the lncRNAs we examined were not examined previously. Aplysia 18S rRNA gene was used to normalize the gene expression levels. A list of primers used in the study is included in Table S2.

In line with the RNAseq findings, our analysis revealed significant changes in gene expression following LTS. Specifically, lncRNA_un9252 exhibited approximately a 3.8‐fold increase (*p* < 0.01) compared to control and a roughly threefold increase (*p* < 0.05) compared to STS (Figure 2k; Table S2). Conversely, lncRNA_un3940 and lncRNA_un7369 displayed approximately 2.8‐ and sixfold decreases, respectively, compared to control. Figure 2l provides a summary of the gene expression levels of the selected mRNA candidates. Further analyses indicated that following LTS training, KDM6A showed approximately a sixfold upregulation (*p* < 0.05) compared to control, while sRNA 2'‐O‐MTase exhibited approximately a 2.8‐fold downregulation (*p* < 0.05) compared to control (Table S2). Importantly, we observed a significant increase of approximately 2.7‐fold (*p* < 0.01) in CREB levels, a well‐known transcriptional activator crucial for learning and LTM (Bartsch et al., 1998). These successful validations through independent experiments lend further support to the notion that both STS and LTS training elicit specific alterations in lncRNA and mRNA expressions in L7MN. Collectively, these findings imply that learning entails the recruitment of distinct changes in the expression levels of key regulators of transcription and RNA processing in L7MN.

### 2.4 DEG analysis of L7MN from 10‐ and 12‐month‐old animals indicates impairments in transcriptional plasticity

To assess the transcriptomic underpinnings of the aging‐associated decline in LTS, we explored the notion that transcriptional plasticity—defined as a cell's ability to undergo specific changes in transcriptome to mediate particular physiological responses—might be compromised with age. This impairment could manifest as either broad alterations across the entire transcriptome or aberrant changes in specific components. Therefore, we next analyzed the transcriptome of L7MN from 10 months (Age 2) and 12‐month‐old (Age 3) animals.

**FIGURE 3:**
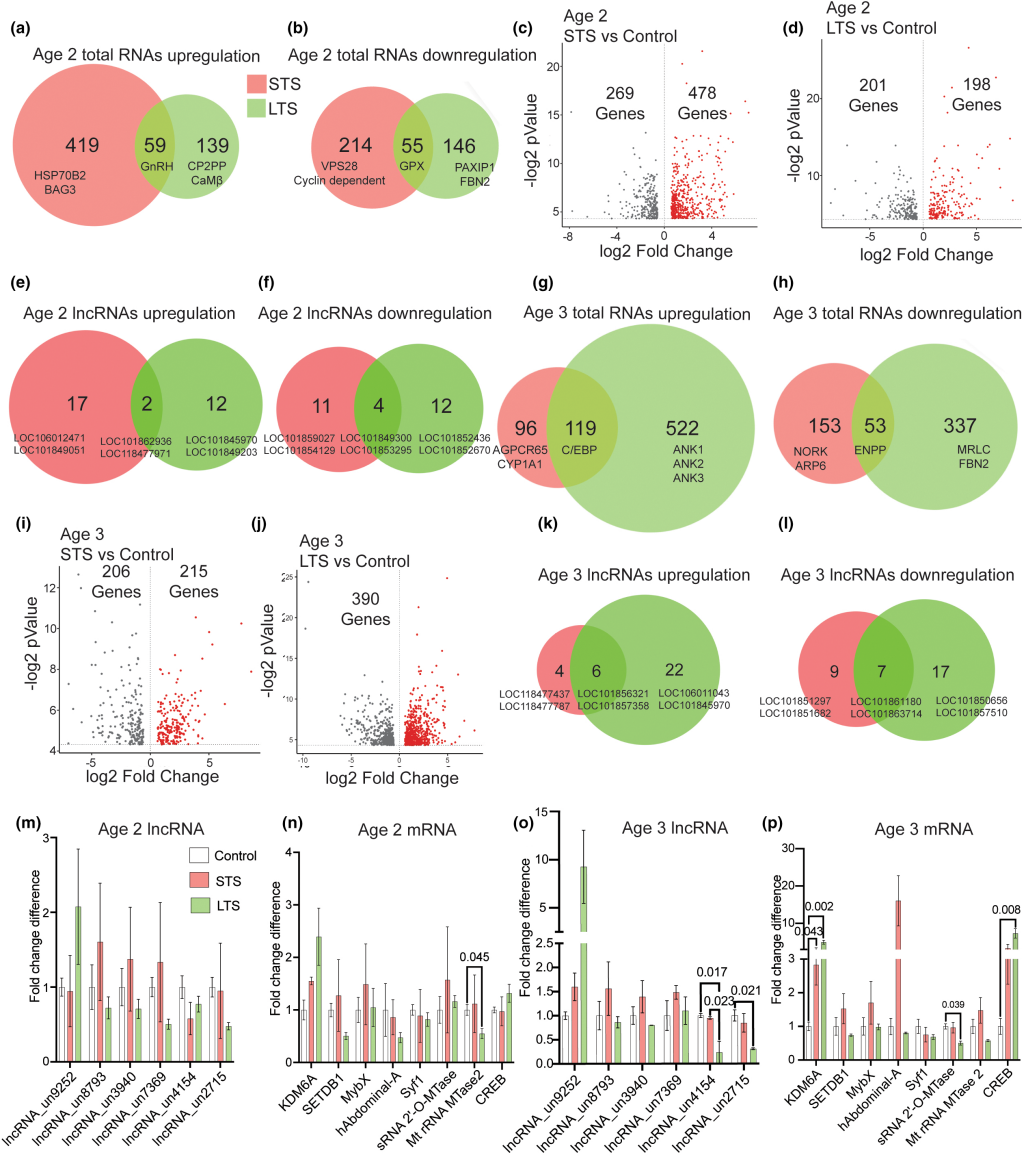
RNAseq analysis of L7MN from 10‐ and 12‐month‐old Aplysia following STS and LTS training. Venn diagram showing Age 2 DEGs (a) upregulated (b) downregulated in response to STS and LTS (*p* <0.05). Differentially expressed genes are ranked in a volcano plot according to their statistical‐log2 *p*‐value (*y*‐axis) and their relative abundance ratio (log2 fold change) between up‐ and downregulated (*x*‐axis). Red dots indicate significantly regulated genes (false discovery rate, <0.01; s0 = 1; *p* <0.05) (see Table S3). Volcano plots of (c) age 2 Control versus STS DEGs, (d) age 2 Control versus LTS DEGs. Venn diagram showing DEG lncRNAs (e) upregulated (f) downregulated in response to STS and LTS (*p* <0.05) (see Table S3). Venn diagram showing age 3 DEGs. (g) Upregulated (h downregulated in response to STS and LTS (age 3; *p* <0.05) (see Table S3). Volcano plots of (I) Age 2 Control versus STS DEGs. (j) Age 2 Control versus LTS DEGs (see Table S3). Venn diagram showing DEG lncRNAs (k) upregulated (l) downregulated in response to STS and LTS (age 3; *p* <0.05) (see Table S3). qPCR validation of the RNAseq data (m) Age 2 lncRNAs, (n) Age 2 mRNAs, (o) Age 3 lncRNAs, (p) Age 3 mRNAs. Relative gene expression levels are shown as the mean fold change, with error bars showing the SEM. One‐way ANOVA followed by Tukey's post hoc test. *N* = 4, *p* values are shown in the bar graphs (see Table S3).

In Age 2 animals, we identified 1317 transcripts, including 52 lncRNAs, while in Age 3 animals, we found 1460 transcripts, including 75 lncRNAs (Figure 3a‐l; Table S3). Analysis of Age 2 animals revealed 747, 399, and 545 transcripts as differentially expressed between STS versus control, LTS versus control, and LTS versus STS, respectively, covering approximately 2.54%, 1.36%, and 1.86% of the annotated genes. Similarly, in Age 3 animals, we observed 421, 1031, and 382 genes differentially expressed between STS versus control, LTS versus control, and LTS versus STS, respectively, encompassing approximately 1.43%, 3.52%, and 1.3% of the Aplysia genome. Figure S7 depicts heatmaps illustrating normalized and scaled expression values of the top 50 differentially expressed genes across training conditions (control, STS, LTS) in both Age 2 and Age 3 groups.

In Age 2 group during LTS, among the upregulated DEGs with synapse signaling were brain‐specific homeobox/POU domain protein 3‐like isoform (LOC101859216), calcium/calmodulin‐dependent 3′,5′‐cyclic nucleotide phosphodiesterase 1C (LOC106014171), GTP‐binding protein RAD (LOC106014213), synapse‐associated protein 1 (LOC101862979), potassium voltage‐gated channel subfamily H member 1 (LOC101860035), FMRFamide receptor (LOC101850131); transcription/translation factors such as myb‐like protein A (LOC101847950), transcription factor MYB120 (LOC101858259), zinc finger A20 and AN1 domain‐containing stress‐associated protein 9 (LOC106013912), forkhead box protein biniou (LOC101856898), Krueppel‐like factor 8 (LOC101850318). In Age 3 group during LTS, we observed synaptic proteins such as cyclic AMP‐dependent transcription factor ATF‐7 (LOC101856191), serine/threonine‐protein kinase fray2 (LOC106012574), cAMP responsive element‐binding protein (LOC100861465), pannexin 2 (LOC100533356), ankyrin repeat domain‐containing protein 17 (LOC101846593), metabotropic glutamate receptor 3 (LOC101863041), serine/threonine‐protein kinases D1, fray2, pakF, RIO1, SIK3 (LOC101864159, LOC106012574, LOC101854275, LOC101845822, LOC101849046) etc.; transcription factors such as forkheadbox protein K2 and O (LOC101847706, LOC101847009), zinc finger protein 16, 271, 493, 628, 704, 708 (LOC101848424, LOC101851507, LOC101852427, LOC118477209, LOC101845787, LOC101860932, LOC101857232), transcription factor 20, MafF, Sox‐10, Sp3, TFIIIB (LOC101853672, LOC101851728, LOC101847270, LOC101863331, LOC101855811). Interestingly we identified several epigenetic regulation related genes such as histone acetyltransferase KAT2A isoform (LOC101856257), histone‐lysine N‐methyltransferase ASH1L isoform (LOC101849998), GADD45 (LOC101853028, LOC101846541), KAT8 regulatory NSL complex subunit 1 (LOC101845902), N‐acetyltransferase ESCO2 (LOC101857418), uncharacterized methyltransferase C25B8.09 (LOC101861999), beta‐1,4‐N‐acetylgalactosaminyl transferase bre‐4 (LOC101851752), threonylcarbamoyl‐adenosine tRNA methylthio‐ transferase (LOC101863090).

In the Age 2 group, several downregulated DEGs stand out, including FMRFamide‐related neuropeptides‐like (LOC101851187), voltage‐gated potassium channel subunit beta‐2 (LOC101861756), and synaptotagmin‐1 (LOC101856907). In the Age 3 group, notable downregulated genes include calmodulin (LOC101850552), voltage‐dependent calcium channel subunit alpha‐2/delta‐3 (LOC101847330, LOC101847576), synapsin isoform 2.1 (LOC100533225), and neuronal acetylcholine receptor subunit alpha/beta‐4 (LOC101861149, LOC101845835)—all crucial for synapse function. Overall, RNAseq analysis across the three age groups reveals an aging‐associated decline as well as aberrant changes in specific components of the L7MN transcriptome in senescent animals.

### 2.5 Validation of differential expression of candidate lncRNAs and mRNAs in 10‐ and 12‐month‐old animals

Continuing our investigation into the transcriptomic changes observed in age group 1, we proceeded to validate the RNAseq findings through qPCR analysis of L7MN isolated from 10‐month‐old (Age 2) and 12‐month‐old (Age 3) animals. Figure 3m illustrates that in the Age 2 group, no significant changes in lncRNA levels were detected, indicating an impairment in the modulation of learning‐associated lncRNAs (identified from LTS analysis of age group 1) with aging. However, upon LTS induction, we observed a threefold decrease (*p* < 0.05) in the expression level of Mt rRNA MTase2 compared to untrained L7MN (Figure 3n; Table S3).

In Age 3 animals, consistent with the RNAseq data, we observed approximately a 4.3‐fold (*p* < 0.05) and 3.2‐fold (*p* < 0.05) decline in the expression levels of lncRNA_un4154 and lncRNA_un2715, respectively, upon LTS compared to control animals (Figure 3o; Table S3). Among the analyzed mRNAs in the Age 2 group, KDM6A expression levels were approximately 2.8‐fold (*p* < 0.05) and fivefold (*p* < 0.01) higher upon STS and LTS, respectively, compared to the control expression levels (Figure 3p; Table S3). Furthermore, following LTS, the expression level of sRNA 2‐O‐Mtase declined approximately twofold (*p* < 0.05). In Age 3 animals, CREB levels in L7MN were approximately 6.4‐fold (*p* < 0.01) higher than the control levels, suggesting aberrant changes in its expression during aging. These results indicate a progressive decline in the expression of learning‐relevant lncRNAs and RNAs during aging. Moreover, multiple genes involved in nuclear functions such as transcription and synapse functions such as excitatory synaptic transmission are impacted in L7MN during aging.

### 2.6 Impact of aging on basal gene expression of L7MN

**FIGURE 4:**
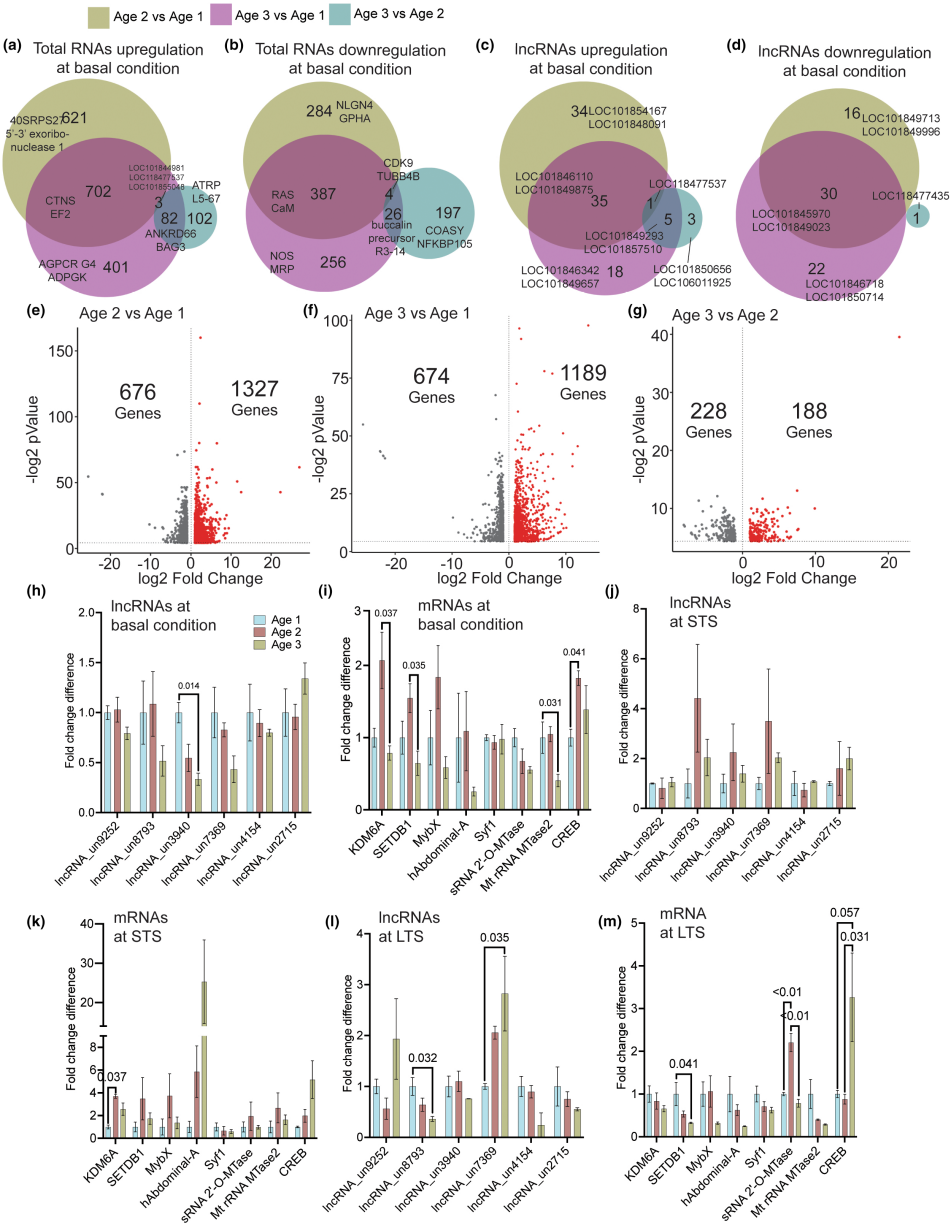
Analysis of aging‐associated changes in L7MN. RNAseq data from untrained animals (used to generate Figures 2 and 3) were independently compared across the three age groups. Venn diagrams showing comparison of upregulated DEGS (a), downregulated DEGs (b), upregulated lncRNAs (c), and downregulated lncRNAs (d) (*p* < 0.05) (see Table S4). (e–g) Differentially expressed genes compared to different age groups are ranked in the volcano plots according to their statistical‐log2 *p*‐value (*y‐*axis) and their relative abundance ratio (log2 fold change) between up‐ and downregulated (*x‐*axis). Red dots indicate significantly regulated genes (false discovery rate, <0.01; s0 = 1; *p* <0.05) (see Table S4). Reanalysis of qPCR candidates from different age groups (see Figures 2 and 3), (h, i) at basal condition, (j, k) in response to short‐term sensitization, (l, m) in response to long‐term sensitization. Relative gene expression levels are shown as the mean fold change, with error bars showing the SEM. One‐way ANOVA followed by Tukey's post hoc test. *N* = 4, *p* values are shown in the bar graphs (see Table S4).

To evaluate how aging affects the basal transcriptome in L7MN, we compared the RNAseq data of untrained animals across the three age groups. Figure 4a–d depicts Venn diagrams illustrating the comparison of total RNA and lncRNAs of up‐ and downregulated differentially expressed genes (DEGs). We observed 1326 upregulated transcripts and 675 downregulated transcripts in Age 2 compared to Age 1 controls; 1188 upregulated transcripts and 673 downregulated transcripts in Age 3 compared to Age 1 controls; and 187 upregulated transcripts and 227 downregulated transcripts in Age 3 compared to Age 2 controls (Table S4). Additionally, Figure 4e–g presents volcano plots displaying the distribution of DEGs (Table S4). These findings collectively suggest that aging compromises the composition of transcriptome in L7MN.

Furthermore, to obtain deeper insights into changes in basal gene expression and understand how aging and learning impact the expression of these candidates across the three age groups, we re‐analyzed the qPCR data (Figure 4h–m; Table S4). At basal condition, we noted a significant ~1.3‐fold decrease (*p* < 0.05) in the expression level of lncRNA_un3940 in Age 3, while KDM6A and SETDB1 levels were found to be increased in the Age 2 group compared to the Age 3 group. Moreover, the level of rRNA methyltransferase 2 in the Age 3 group was significantly lower than in both Age 1 and Age 2 groups, and CREB levels in the Age 2 group were higher than in both the Age 1 and Age 3 groups.

In STS‐trained animals, no significant changes in gene expression were observed among the age groups, except for KDM6A levels, which were significantly higher (~3.7‐fold; *p* < 0.05) in the Age 2 group compared to the Age 1 group (Table S4). Analyzing LTS samples, we observed significantly lower (~2.8‐fold; *p* < 0.05) expression of lncRNA_un8793 in the Age 2 group compared to the Age 1 group. In contrast, lncRNA_un7369 levels in the Age 3 group were significantly higher (~2.8‐fold; *p* < 0.05) than in the other age groups. Among LTS samples, SETDB1 showed a notable decrease (~3.1‐fold, *p* < 0.05) in the Age 3 group, while sRNA 2’‐O‐MTase levels in the Age 2 group were the highest (~2.2‐fold, *p* < 0.01). Additionally, CREB levels in the Age 3 group were ~3.2‐fold higher (*p* = 0.05) compared to the Age 1 and Age 2 groups. These results further confirm that aging leads to aberrant changes in gene expression in L7MN during senescence.

### 2.7 STS and LTS regulated genes in L11 and R2 neurons

**FIGURE 5:**
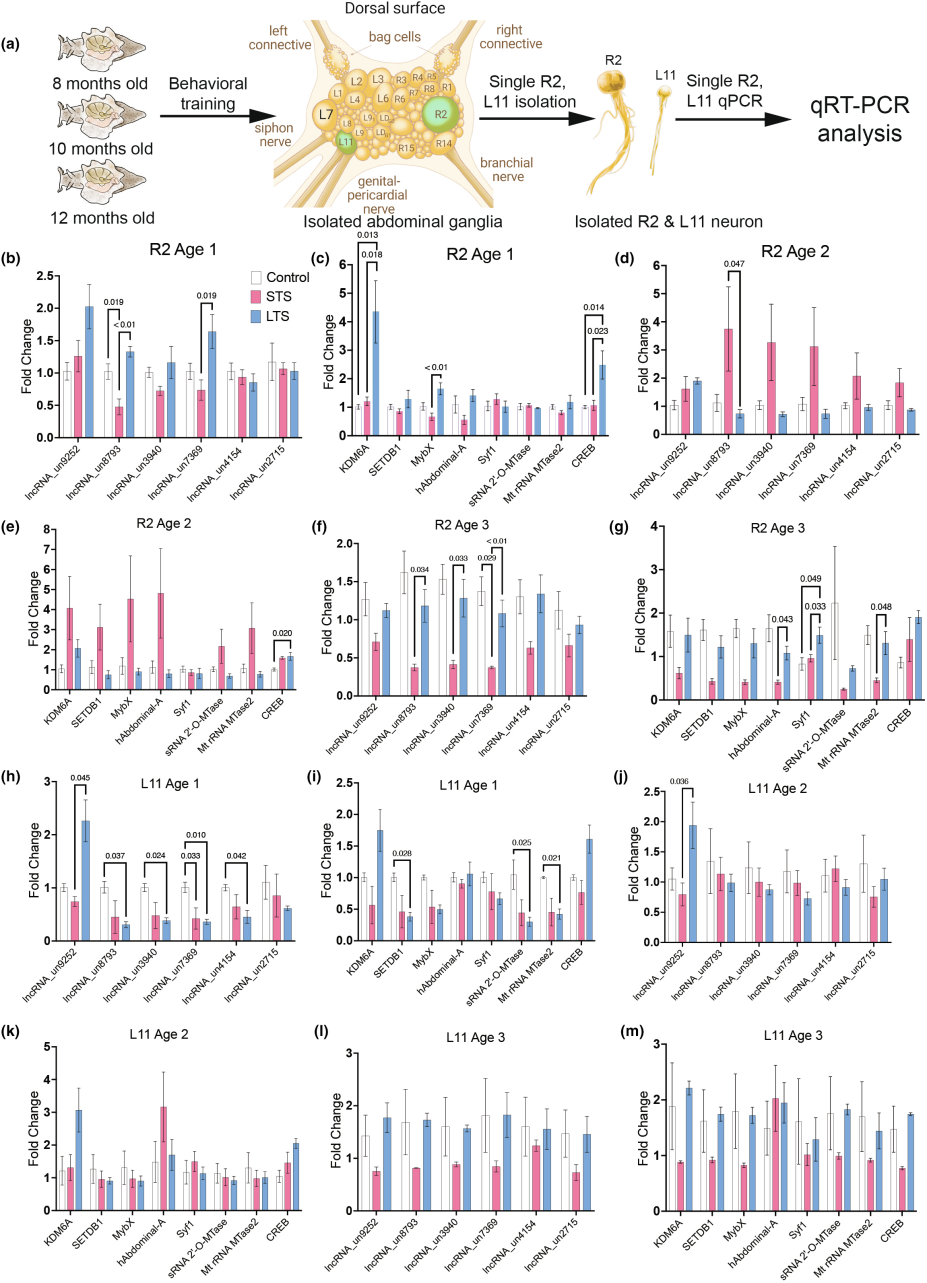
Gene expression analysis of R2 and L11 MN neurons following STS and LTS training. (a) Schematic representation of the workflow for single R2 and L11 neuron isolation and qPCR analysis from trained (STS and LTS), and untrained control Aplysia from the three age groups. (b–g) Analysis of qPCR candidates in R2 across different age groups (see Table S5). (h–m) Analysis of qPCR candidates in L11 across different age groups. Relative gene expression levels are shown as the mean fold change, with error bars showing the SEM. One‐way ANOVA followed by Tukey's post hoc test. *N* = 5, *p* values are shown in the bar graphs (see Table S5).

Our RNAseq analyses, coupled with independent validations in L7MN, highlight widespread transcriptomic changes associated with a decline in LTS during aging. Moreover, these analyses have revealed that STS and LTS distinctly reshape the composition of the transcriptome in L7MN. Motivated by these findings, we turned our attention to investigating how aging, STS, and LTS influence the transcriptome of two other identified neurons in the abdominal ganglia: L11 motor neuron (L11MN) and a giant cholinergic neuron R2 (Figure 5). These neurons were previously not known to be involved in mediating STS or LTS of the SWR. R2, being a giant cholinergic neuron, is considered the largest neuron identified in the animal kingdom and assists in escape locomotion during sensitization in Aplysia (Stopfer & Carew, 1988). Moreover, gene expression in R2 has been shown to be altered with aging (Stopfer & Carew, 1988). On the other hand, L11MN sends projections to the gill and participates in foot contraction and locomotion in Aplysia (Romanova et al., 2007). Therefore, we isolated L11MN and R2 from trained and untrained animals and assessed the expression of selected lncRNAs and mRNAs (see Figure 2) in these neurons by single cell qPCR. Importantly, similar to L7MN, there is only one L11MN and one R2 neuron in the entire animal and they are localized in the abdominal ganglia.

Based on the lack of known involvement of L11MN and R2 in sensitization and aging, we anticipated no change in gene expression following training. However, our single L11MN and R2 qPCR analyses show that in response to STS and LTS expression of genes is altered (up‐ or downregulated) in unique ways (Figure 5; Figures S8 and S9; Table S5). In R2 LncRNA 8793 was altered in both STS (downregulated) and LTS (upregulated), 7369 was upregulated in LTS, and mRNAs KDM6A, MyoX and CREB were altered in age group 1 (Figure 5b,c; Table S5). In age group 2 fewer candidates were altered (lncRNA 8793 and mRNA CREB), but in age group 3, more lncRNAs (8793, 3940, 7369) and mRNAs (hAbdominal A, Syt1, MtrRNA MTase 1) were altered in response to LTS when compared to STS training (Figure 5d‐g; Table S5).

In contrast, L11MN displayed recruitment of multiple lncRNAs (five out of six lncRNAs studied) and mRNAs (three out of eight, with KDM6A and CREB not reaching significance) in age group 1, similar to L7MN (Figure 5h,i; Table S5). However, these changes did not persist (except for lncRNA 9252 in age group 2) in age groups 2 and 3 (Figure 5j‐m; Table S5). Overall, these findings demonstrate that lncRNAs and mRNAs are differentially modulated in a neuron‐specific manner during aging and learning. Furthermore, these results suggest the potential involvement of R2 and L11MN during STS and LTS learning.

### 2.8 Comparative analysis of expression changes in L7, L11, and R2 neurons suggest neuron‐specific modulation of learning relevant genes

Our finding that certain genes modulated in L7MN by STS and LTS are also affected in R2 and L11 prompted us to investigate whether these genes exhibit similar modulation across these neurons. Significant differences in the degree of up‐ or downregulation of these candidate genes would suggest neuron‐specific regulatory mechanisms. Thus, we conducted a comparative analysis of the magnitude of fold changes in these neurons by re‐examining the qPCR data. The data were normalized to the corresponding 18 s rRNA levels in each neuron (L7MN, L11MN, and R2). This analysis across the three neurons revealed several lncRNAs and mRNAs that are significantly enriched in these neurons at basal levels as well as in response to STS/LTS training.

For instance, lncRNA 3940, downregulated in L7MN in response to STS and LTS, was found to be enriched in L7MN compared to R2 and L11MN under basal conditions. Similarly, lncRNA 9252, upregulated in L7MN in response to LTS, exhibited enrichment in L11MN and was significantly enriched in L7MN compared to L11MN. Notably, the expression of hAbdominal‐A mRNA was enriched in R2 compared to L7MN and L11MN, while SETDB1 was enriched in L7MN compared to L11MN and R2. Additionally, CREB was enriched in both L7MN and L11MN compared to R2 in age group 1 (Figures S10–S12; Table S5).

### 2.9 Identification of lncRNA–mRNA associations modulated by LTS

Our observation indicating that expression of multiple lncRNAs are altered in L7MN during learning and aging prompted us to examine potential *cis*‐regulated target RNAs among the DEGs. Furthermore, expression, regulation and function of lncRNAs remain poorly understood in Aplysia. Nuclear enriched lncRNAs could potentially modulate expression of RNAs within 200 Kb of their locus in the genomic region (Raveendra et al., 2018). This regulation is described as *cis*‐regulation of lncRNA targets and involves lncRNA interacting with transcriptional machinery mediating transcription of specific targets in proximity. Importantly, considering the complex ways by which transcription is modulated, *cis*‐regulation mediated by specific lncRNAs could be context specific.

**FIGURE 6:**
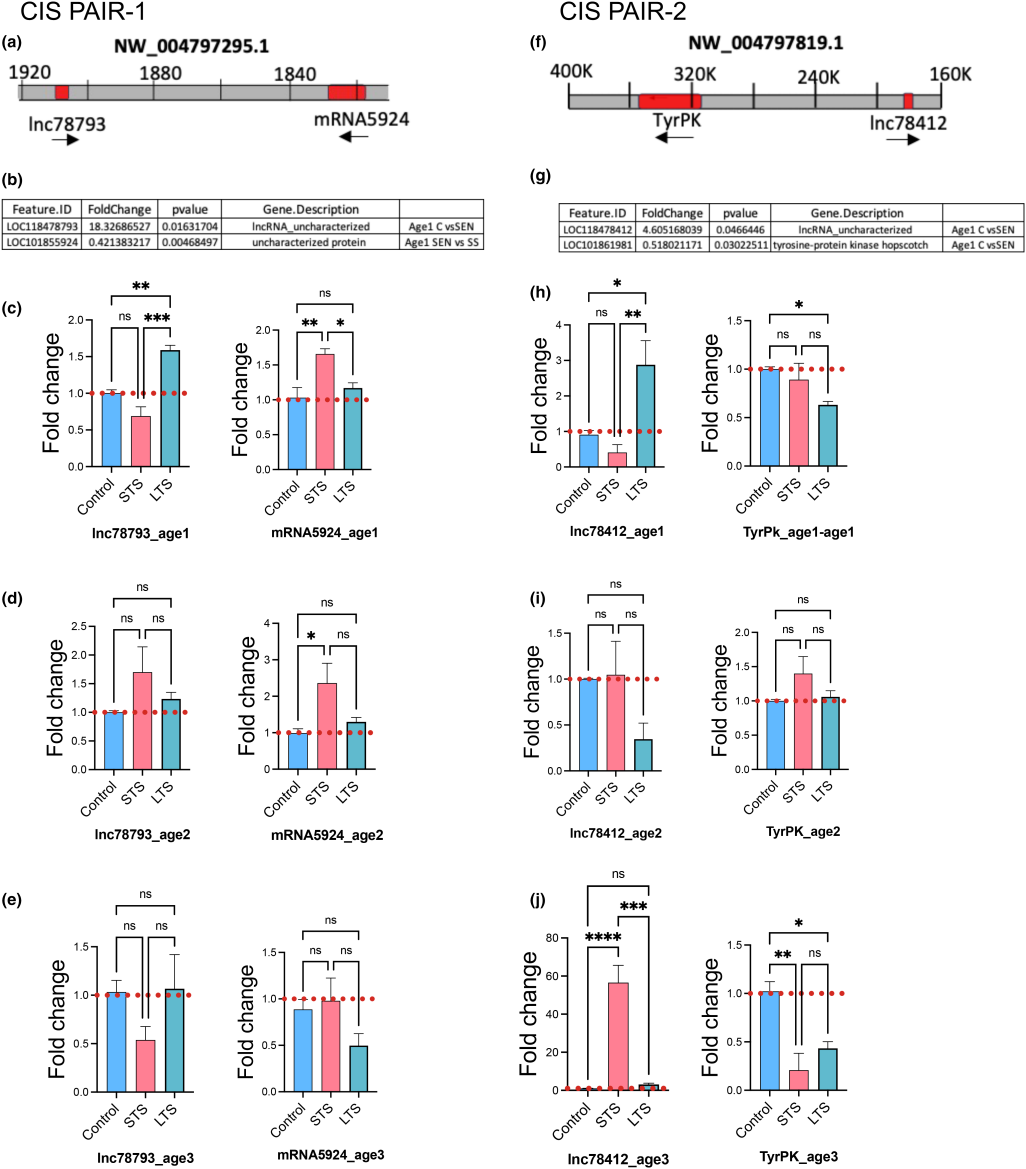
Validation of RNA seq data by qPCR for lncRNAs and its cis pair (± 200 kb). Two lncRNAs and their protein coding mRNAs cis pairs from age1 RNA Seq are examined. Validation of the RNA seq data were done by qPCR for STS and LTS for all three age groups. (a–f) Schematic representation of the location of lncRNAs and its mRNA pair in the genomic region for Cis Pair1 and 2. (b, g) RNASeq data of Cis pair1 and 2 for age1 The Cis pair 1 is non coding RNA lnc78793 (LOC118478793) and uncharacterized protein coding gene mRNA5924 (LOC 101855924) as its Cis pair1. The Cis pair‐2 is non coding RNA is lnc78412 (LOC118478412) and mRNA of tyrosine‐protein kinase hopscotch (LOC101861981) as its cis pair. (c–j) qPCR data of the relative expression of lncRNAs and its cis pair for STS and LTS compared to control for Age 1 (c, h), Age 2 (d, i) and Age 3 (e, j) (see Table S6).

We first assessed the expression of lncRNAs in nucleus versus cytoplasm fractions and then identified potential regulated RNAs within 200 kb of their loci, based on bioinformatics prediction. We next searched DEGs to assess whether these potential RNA targets are present among the DEGs (Figure S13). Our qPCR assessments of top 25 lncRNAs in L7MN (Table S6) selected based on L7MN RNAseq revealed 10 lncRNAs enriched in the nucleus (Figure S13). Further analysis of RNAseq dataset identified several lncRNA and their potentially *cis*‐regulated targets among the DEGs [lncRNA_78793] (~4.2‐fold upregulated; Age 1 group LTS): uncharacterized proteins LOC101855924 showed ~1.2‐fold downregulation (Age1 LTS vs. STS); lncRNA_0492 (~3.2‐fold upregulated, Age 2 group LTS): TBC1 domain family member 13 is ~0.66‐fold upregulation (Age2 LTS vs. STS) lncRNA_7178 (~2.57‐fold downregulated, Age 2 LTS): Mucin‐5 AC (LOC10185663) and golgin subfamily A member 3 (LOC101846517) showed ~1.3‐fold and 0.63‐fold downregulation (Age2 LTS vs. CTRL and Age2 LTS vs. STS); lncRNA_3167 (~2.02‐fold downregulated, Age 2 LTS downregulated): Uncharacterized protein LOC101856926 (LOC101856926) and histone H2A‐like (LOC101855053) show ~1.4 and ~1.5‐fold downregulation (Age2 LTS vs. CTRL and Age2 LTS vs. STS) (Table S6). Importantly regulation of these predicted lncRNA‐mRNA pairs were not identified to be modulated in age group 3. Thus, these observations suggest that specific lncRNA‐mRNA pairs are recruited during learning but that recruitment is impaired during aging in L7MN. We next focused on two lncRNA‐mRNA pairs (Figure 6a,f) and assessed whether their regulation during learning follow bioinformatics prediction. As indicated in Figure 6b, based on RNAseq data lncRNA 78793 expression was significantly upregulated in LTS trained neurons when compared to untrained controls. However, its predicted cis‐target mRNA 5924 was significantly downregulated when LTS trained and STS trained neurons were compared. Our independent single cell qPCR experiments confirm these findings in age group 1 (Figure 6b,c). This regulation was impaired in age groups 2 and 3 (Figure 6d,e). Similarly, lncRNA 78412 is significantly upregulated following LTS training when compared to untrained control in age group 1. Its predicted cis mRNA target protein tyrosine kinases (TyrPk) show significant down regulation of expression in the same comparisons (Figure 6g). Figure 6h shows independent validation of RNAseq data and prediction. This regulation was impaired in age group 2 (Figure 6i). However in age group 3, we find that the expression of this lncRNA was significantly upregulated in STS trained neurons, contrary to what we observed in age group 1. The expression of TyrPk also shows dysregulation, with increase in the lncRNA resulting in decrease in the expression of mRNA (Figure 6j). Taken together, our findings suggest that specific lncRNA‐mRNA pairs are recruited during learning but that recruitment is impaired during aging in L7MN, emphasizing the dynamic interplay between lncRNAs and their targets in neural plasticity and age‐related changes.

## 3 DISCUSSION

Numerous studies have indicated broad transcriptome‐wide alterations associated with aging (Azam et al., 2021; Foster, 2002; Mattson & Arumugam, 2018; Rizzo et al., 2014; Schimanski & Barnes, 2010). However, the specific impact of aging on the expression of genes relevant to neuronal plasticity in the context of LTM remains poorly understood. Consequently, elucidating the changes induced by learning in distinct components of the transcriptome becomes a crucial step in comprehending the consequences of aging on LTM (Alberini & Kandel, 2014; Lee et al., 2008; Yap & Greenberg, 2018). The identification of transcriptional signatures linked to both aging and learning holds the potential to pave the way for the development of therapeutics tailored to the subpopulation of neurons most susceptible to impairments during aging. In our study, we focused on a pivotal neuron (L7MN) engaged in two temporally distinct nonassociative learning paradigms, STS and LTS, aiming to assess the influence of aging on transcriptional plasticity.

Our approach successfully pinpointed changes induced by STS and LTS in both the long‐noncoding and coding transcriptome of L7MN. Overall, comparison of the three age groups, gene expression changes showed a progressive decline as well as aberrant changes in the expression in age group 3 (senescent animals). The comparison of STS versus untrained control showed a decline in gene expression (group1 vs. group 2 vs. group 3: 1364 vs. 1188 vs. 763 DEGs). The comparison of LTS versus control showed an increase in the downregulated DEGs and decrease in the upregulated DEGs in age group 2 (group 1 vs. group 2: downregulated: 276 vs. 348; upregulated: 584 vs. 568) whereas group 3 showed increase in both upregulated and downregulated DEGs (group 1 vs. group 3: upregulated: 584 vs. 974; downregulated: 276 vs. 627 DEGs) indicating aberrant gene expression changes in senescent animals. Comparison of LTS versus STS showed progressive decline in DEGs (group1 vs. group 2 vs. group 3: 1235 vs. 944 vs. 687 DEGs). Together, these analyses indicate the transcriptional landscape of L7MN undergoes complex changes during learning and aging. To obtain deeper insights into this complex regulation, we next assessed the biological functions of DEGs. While all the DEG lncRNAs identified are currently uncharacterized, the DEG mRNAs are implicated in mediating transcriptional, translational, cytoskeletal, and synaptic functions. These findings align with earlier observations regarding aging‐associated neuronal changes (Azam et al., 2021; Foster, 2002; Mattson & Arumugam, 2018; Rizzo et al., 2014; Schimanski & Barnes, 2010). For the independent validations, across all age groups we focused on single neuron qPCR analysis of eight genes that functions as critical modulators of transcription and RNA processing.

Importantly, genes differentially modulated by STS and LTS include those involved in epigenetic and transcriptional regulation (examples include KDM6A and CREB), indicating that unlike STS, LTS induces enduring changes in the transcriptional landscape of L7MN. Examination of STS and LTS modulated genes across the three age groups suggested aberrant expression with age. Specifically, in age groups 2 and 3, both STS and LTS‐induced changes in KDM6A were absent, whereas in age group 2, CREB regulation was absent. In contrast to CREB regulation in age group 1, there was no difference between STS and LTS in modulating CREB in age group 3, supporting the lack of sensitization in age group 3.

Previous studies have extensively documented changes in lncRNA expression associated with aging (Marques & Ulitsky, 2019; Pereira Fernandes et al., 2018; Qi et al., 2022). However, the modulation of aging‐specific lncRNAs relevant to learning processes remains poorly understood. In our investigation, we observed significant alterations in the expression of specific lncRNAs and their predicted target genes in response to LTS training. Notably, our analysis revealed that LTS‐induced changes in lncRNAs exhibited deficits in their expression during the aging process. Crucially, in age group 3, modulation of predicted lncRNA‐mRNA pairs was notably absent, distinguishing it from other age groups. Collectively, these findings highlight deficits in transcriptional plasticity during aging.

Enquiring about the specificity of STS and LTS‐induced changes in the transcriptional landscape of L7MN, we sought to explore whether genes influenced by STS/LTS in L7MN are also modulated in two other neurons—L11MN and R2—that do not have known functions in STS or LTS of siphon withdrawal. Our qPCR analysis of candidate genes (both lncRNAs and mRNAs identified in L7MN) suggested transcriptional modulation of specific genes by STS/LTS in both L11 and R2. Notably, these changes were more pronounced in L11MN compared to R2.

It has been demonstrated that L11MN are subject to regulation by both paracrine and autocrine diffusible factors from sensory neurons following synapse formation (Alexandrescu & Carew, 2020). Additionally, L11MN plays roles in Aplysia foot contraction and locomotion (Romanova et al., 2007). Although the possible role of L11MN in LTS remains elusive, the discovery that L11MN could be modulated by diffusible factors suggests a potential role for its gene expression changes in LTS. It is possible that the activation of sensory neurons and interneurons during LTS training produces diffusible factors that modify signaling in L11MN, though this needs experimental validation. The observed transcriptional changes in L11MN and R2 in response to STS and LTS training imply potential roles for these neurons in mediating LTS. Importantly, the analysis reveals neuron‐specific changes during LTS, such as lncRNA 9252 and 7369 in both L7MN and L11, as well as KDM6A and CREB in L7MN and R2. In age group 3, all three neurons exhibit either a complete lack of alteration (including both upregulation and downregulation of specific transcripts), suggesting severe impairments in transcriptional plasticity in senescent animals.

Significantly, all three neurons are situated in the same ganglion—the abdominal ganglion of Aplysia's central nervous system—yet they display different trajectories of aging. These findings underscore that aging‐induced changes may not be identical across all cell types, aligning with previous research (Allen et al., 2023; Kadakkuzha et al., 2013; Moroz & Kohn, 2010; Ximerakis et al., 2019). Collectively, our data emphasize the importance of single neuron and neural circuit‐based assessments of aging to pinpoint specific deficits induced by aging. We identified both qualitative and quantitative changes in the coding and noncoding transcriptomes during aging, highlighting impairments in transcriptional plasticity, and a cell‐specific manner of aging that likely underpins aging‐associated cognitive decline. While we validated a subset of transcriptional changes, numerous lncRNAs and mRNAs remain to be characterized. Although our work illuminates transcriptomic correlates of aging‐associated impairments in LTS, future studies are needed to assess the functions and mechanisms of differentially regulated lncRNAs and mRNAs, as well as the possible role of L11MN and R2 in LTS.

Collectively, our study offers novel insights into the transcriptional landscape of L7MN and sheds light on how learning and aging intersect in this context. The data presented in this manuscript are expected to serve as a valuable resource for the neuroscience community and those studying the biology of aging and learning. For instance, our lncRNA data could facilitate future studies aiming to determine the role of the noncoding transcriptome in modulating plasticity and aging. Integrating these studies with functional assays may reveal how noncoding and coding transcriptomes interact for neuronal plasticity and how aging impacts their interaction and function. Understanding how transcriptomic changes in individual neurons modulate specific learning and LTM is vital for obtaining novel mechanistic insights into aging‐associated cognitive decline and developing therapeutics.

## 4 MATERIALS AND METHODS

### 4.1 Animals

#### 4.1.1 Aging cohorts

Two cohorts of animals were reared and maintained at the National Resource of Aplysia at the University of Miami's Rosenstiel School of Marine, Atmospheric, and Earth Science. Cohort one (Group 1) hatched on February 2, 2019. Cohort 2 (Group 2) hatched on March 1, 2019. Animals were reared at 15°C and fed red algae ad libitum before training began. Behavioral training, including sensitization with one or four tail shocks (Pinsker et al., 1973; Sadhu et al., 2023) and no shock controls was performed on animals at 8 (Age 1), 10 (Age 2), and 12 months (Age 3) of life.

#### 4.1.2 Before the pretest

Thirty animals from each cohort were selected for each age group to investigate age‐related memory deficits in *Aplysia*. If possible, active animals with similar body sizes were chosen for training (Table S1). Because the SWR was used to measure long‐term memory capacity, animals with larger siphons were preferred. Animals were selected for training based on appearance, weighed, and placed in individual cages for 1 week before the pretest. Algae access was restricted 3 days before the pretest. Observations such as egg mass formation, abnormal locomotion, animal physical appearance, and weight were noted throughout the experiment. *Aplysia* body mass increases during development but declines after sexual maturity and aging.

#### 4.1.3 Behavior training short‐term and LTS

*Pretest*: On day 1 of training, a paintbrush bristle was used as a non‐noxious stimulus (touch) to elicit siphon withdrawal. The duration of siphon withdrawal from the time of the stimulus to the beginning of relaxation was recorded by a blind observer. Each animal received four touches. The animal's average SWR or pretest value was calculated and used to group the animals for training so that each group's average SWRL pretest value was similar (Table S1).

*Behavioral training*: Five groups were used to investigate age‐related learning deficits: two groups for behavioral measurements (B) following four shocks for LTS or no shock control, and three groups for single‐cell isolation (SCI) and RNA analysis following either one shock for STS, four shocks for LTS, or no shock control. Day 2 of training included mock tail shocks for the control groups and either a single tail shock (STS training) or four tail shocks (LTS training) separated by 30 min for the sensitization groups (Antonov et al., 1999; Frost et al., 1985). Each tail shock consists of four trains, each with a duration of 1500 ms, with a shock rate of 0.33 pulse per second (PPS). For the SCI groups, animals' abdominal ganglia were dissected 1 h after training, and single neurons (L7, L11, and R2) were isolated for RNA analyses.

*Test*: On day three, the behavioral groups' long‐term memory was tested. Four siphon touches were elicited, and the average SWRL was compared to the average pretest value as a measure of training retention.

### 4.2 Isolation of L7MN, L11MN, and R2 for RNA extraction

To investigate the transcriptional dynamics at a single neuron level, we isolated the L7MN, L11MN and R2 neurons from the abdominal ganglia from the STS and LTS‐trained sea slugs. Following 1 h after the last shock, the abdominal ganglia were dissected from the animals and single neurons were collected as described by (Akhmedov et al., 2013). The total RNA was extracted using the Arcturus™ PicoPure™ RNA Isolation Kit (Applied Biosystems), and subjected to total and small RNA sequencing. See Table S1 for details of the batch, and neurons used for RNA isolation and analysis.

One hour after behavior training, Aplysia were injected with isotonic MgCl_2_ for 5–10 min (equivalent to 30%–35% of the animal's body weight). Following the methodology protocol from Akhmedov et al. (2013), the abdominal ganglia (with long, intact L. and R. connective nerves, and as long as possible siphon, genital‐pericardial and branchial nerves) were isolated and treated with 0.1%–0.3% protease in artificial seawater (ASW) for ~1–2 h, depending on body weight. After digestion, ganglia were pinned in a Sylgard Silicone chamber and perfused with ASW, desheathed, and the target neurons were identified as described in Akhmedov et al. (2013). Areas around the L7, L11, and R2 neurons were cleared, and neighboring neurons were removed to ensure the isolation of single cells. Then, 100% ethanol was perfused over the ganglia to petrify the neurons, and target neurons were individually isolated with forceps and placed along the wall of a frozen nonstick 1.7 mL microfuge tube on dry ice.

The axon length and thickness of R2, L7, and L11 differ. R2 axons project from the left abdominal ganglion to the ipsilateral, contralateral pedal, and pleural ganglia (Moroz & Kohn, 2010). The L7 axons project to the siphon, genital‐pericardial, and branchial nerves (Leonard & Edstrom, 2004). L11 has many branches from its axons. While each target neuron's average thickness and length are unique, we established a grading system (a–e) to classify the length of the axons from each isolated neuron (Table S1). Neurons with relatively exceptionally long axons were an “A.” Neurons with relatively long axons were classified as “B.” Neurons with short axons were graded as a “C.” Neurons with a small segment of axon were considered “D.” Lastly, neurons with only the cell body isolated were considered “E.” Only L7 “A” and “B” isolated neurons were considered for RNA sequencing.

### 4.3 RNAseq analysis

RNAs were isolated from single L7MN using the Arctus LCM RNA isolation kit, and the quality of RNAs was assessed using a bioanalyzer. We obtained 20 ng/μL RNAs (total 10 μL eluted RNA from one microdissected cell body) that we used for RNAseq (Clontech SMART‐Seq Ultra Low Input RNA kit) in Scripps Florida Genomics Core (Currently known as The Herbert Wertheim UF Scripps Institute for Biomedical Innovation & Technology). After removal of ribosomal RNAs using a custom kit developed at the Scripps Genomics Core, RNAs were sequenced using Hiseq500. In this experiment, we obtained ~20 million reads per sample (*n* = 4–6 for each condition). After quality control, the reads were mapped to the Aplysia genome (seahare‐NCBI‐aplcal3.0).

RNAseq analyses were carried out by Maryland Genomics, Institute for Genome Sciences, UMSOM. Illumina libraries are mapped to the *A. californica* reference, NCBI RefSeq accession GCF_000002075.1, using HiSat2 v2.0.4, using default mismatch parameters. The read counts for each transcript are generated by HTseq, the reads are either normalized as CPM (count per million reads), or RPKM (Reads Per Kilobase Million). The DESeq2 Bioconductor package (v1.5.24) is used to estimate dispersion, to normalize read counts by library size, to generate the counts per million for each gene, and to determine differentially expressed genes between experiment and control samples. Figures S2–S6 show sample clustering and alignment summary. The alignments were generated by HISAT2. Samtools was used to generate alignment statistics. Differentially expressed transcripts with a raw *p* ≤ 0.05 and a minimum 1.5× fold change between groups were used for downstream analyses. RNAseq was deposited to NCBI and can be accessed (GEO accession number: GSE234983).

### 4.4 Quantitative real‐time PCR

Following our previously stated protocols (Badal et al., 2019; Liu et al., 2014; Sadhu et al., 2023) quantitative real‐time PCR (qRT‐PCR) analyses were conducted to validate the RNAseq data. One hour after behavioral training, L7, L11, and R2 neurons were collected from the abdominal ganglia as described in the previous section from all age groups. Using the Arcturus™ PicoPure™ RNA Isolation Kit, total RNA was extracted from the single neurons individually, and cDNA was prepared using qScript cDNA supermix. Aplysia 18S rRNA reference gene is used for normalization. Relative quantification of each transcript was done following the 2^−ΔΔCt^ method (Livak & Schmittgen, 2001).

### 4.5 lncRNA target analysis

To analyze the cytoplasmic or nuclear localization of the lncRNAs detected (Table S6) in the RNAseq experiment, cytoplasmic or nuclear fractionation of RNA was isolated from the abdominal ganglia using Norgen Biotek Corp Cytoplasmic & Nuclear RNA Purification Kit following the manufacturer's protocol. cDNAs were generated from the purified RNA using qScript cDNA supermix were used in the qPCR analysis. We next focused on nuclear enriched lncRNAs and searched for potential RNAs transcribed 200 kb upstream or downstream of the loci of candidate lncRNAs (potential *cis*‐regulated RNAs) by manually searching *Aplysia* genome sequences. We then selected predicted *cis*‐targets and examined whether they are among the DEGs identified from the RNAseq data from L7MN. lncRNAs identified from RNAseq and also within 200 kb of lncRNA loci were considered as potential targets of candidate lncRNAs.

### 4.6 Statistical analyses

Statistical analyses were conducted in R and Prism 9. All data used for preparing Figures and corresponding statistical analyses are available in the Table file. Behavior data was analyzed by using a three‐way ANOVA followed by individual post hoc comparisons. qPCR data was analyzed by using one‐way ANOVAs followed by post hoc tests unless indicated otherwise. The results are graphically represented as the mean ± standard error of the mean (SEM) throughout the text, unless otherwise stated. *N* represents the number of independent samples for each experiment. All data used for preparing figures and statistical analyses are included in supplementary tables.

## AUTHOR CONTRIBUTIONS

SVP designed the project with inputs from RDH. PG and DS help set up aging cohorts and maintained them. LF and MCS provided all infrastructural resources and guidance regarding setting up aging cohorts. KB optimized single neuron isolations, carried out all behavior analyses, prepared and interpreted behavior data, and isolated L7MN, L11MN, and R2 neurons. AS carried out all L7MN qPCR analyses and prepared all figures. BLR and SLV analyzed R2 and L11 neurons by qPCRs. CM conducted all bioinformatics analyses with help from AS and AM. SVP, AS and BLR interpreted results. SVP and AS wrote the paper. SP and RDH revised the manuscript based on inputs from authors.

## FUNDING INFORMATION

This information is included in the acknowledgements. NSF (Grant 1453799) to SVP, NIH (1R21AG055049, 1R01MH119541 and 1R01MH118444) to SVP, 1R01NS113903 to RDH, 1F31MH127958‐01A1 to KKB, and P40OD010952 to MCS.

## CONFLICT OF INTEREST STATEMENT

The authors declare no conflicts of interests.

## Supporting information


Data S1.

## Data Availability

All the RNAseq data are available from NCBI (accession number: GSE234983).
