# Supplementary material for: Single‐neuron analysis of aging‐associated changes in learning reveals impairments in transcriptional plasticity
Source: Aging Cell. 2024 Jun 24;23(9):e14228. doi: 10.1111/acel.14228 (PMC11488329; doi:10.1111/acel.14228)
Supplement: Supplementary file 1 — Data S1. [file ACEL-23-e14228-s001.zip › Badal et al Supplementary Information-V6.pdf]

Single neuron analysis of aging associated changes in learning reveals impairments in transcriptional plasticity

Kerriann K Badal[1,2], Abhishek Sadhu[#1], Bindu L Raveendra[#1],Carrie McCracken[3], , Sebastian Lozano-Villada[11,,4], Amol C Shetty[3], Phillip Gillette[5], Yibo Zhao[1], Dustin Stommes[5], Lynne A Fieber[5], Michael C Schmale[5], Anup Mahurkar[3], Robert D Hawkins[6,7], Sathyanarayanan V Puthanveettil[1*]

[1]Department of Neuroscience, The Herbert Wertheim UF Scripps Institute for Biomedical Innovation & Technology, 130 Scripps Way, Jupiter, FL 33458, USA

[2]Integrated Biology Graduate Program, Florida Atlantic University, Jupiter, FL 33458, USA

[3]The Institute for Genome Sciences, University of Maryland School of Medicine, Baltimore, MD, USA

[4]Harriet L. Wilkes Honors College, Florida Atlantic University, 5353 Parkside Drive, Jupiter, FL 33458, USA

[5]National Resource for Aplysia, University of Miami Rosenstiel School of Marine, Atmospheric, and Earth Sciences, 4600 Rickenbacker Causeway, Miami FL 33149, USA

[6]Department of Neuroscience, Columbia University, New York, New York 10032, USA
[7]New York State Psychiatric Institute, New York, New York 10032, USA

# Contributed equally

*Corresponding author email address: sputhanveettil@ufl.edu

Abbreviated title: “Transcriptomic bases of aging associated memory decline”

Number of Supplementary Figures: 13

Number of Supplementary Tables: 6

Supplementary Figures

Supplementary Figure S1

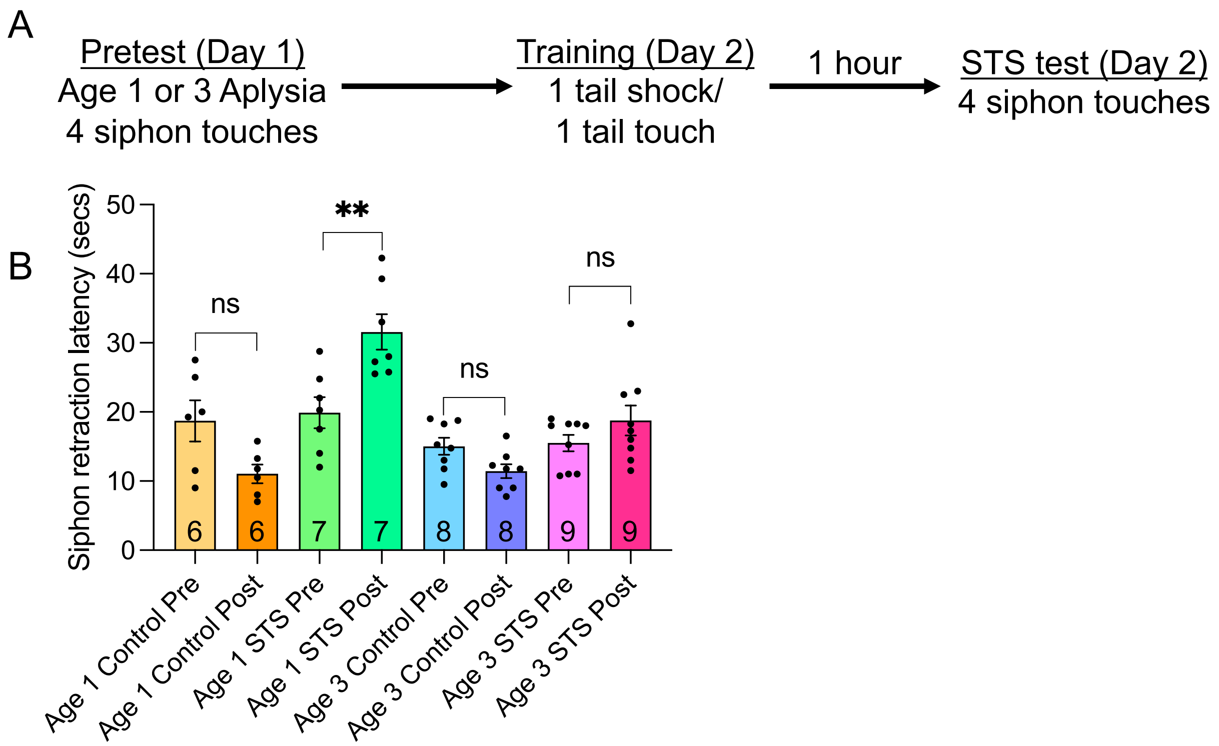

Supplementary Figure 1. Short-term sensitization in Ages 1 and 3. A. Schematic of Short-term sensitization (STS) training in age groups 1 and 3 **B.** Bar graphs showing the average duration of siphon withdrawal from the stimulus to the time the siphon begins to relax before (Pre) and after (Test, 1 hour after) STS training in age groups 1 and 3. The number of animals used for analysis is shown in the bar graphs. **p-Value<0.001, NS: non-significant. One Way ANOVA followed by Tukey's post hoc test, Error bars are SEM, (see Supplementary Table S1).

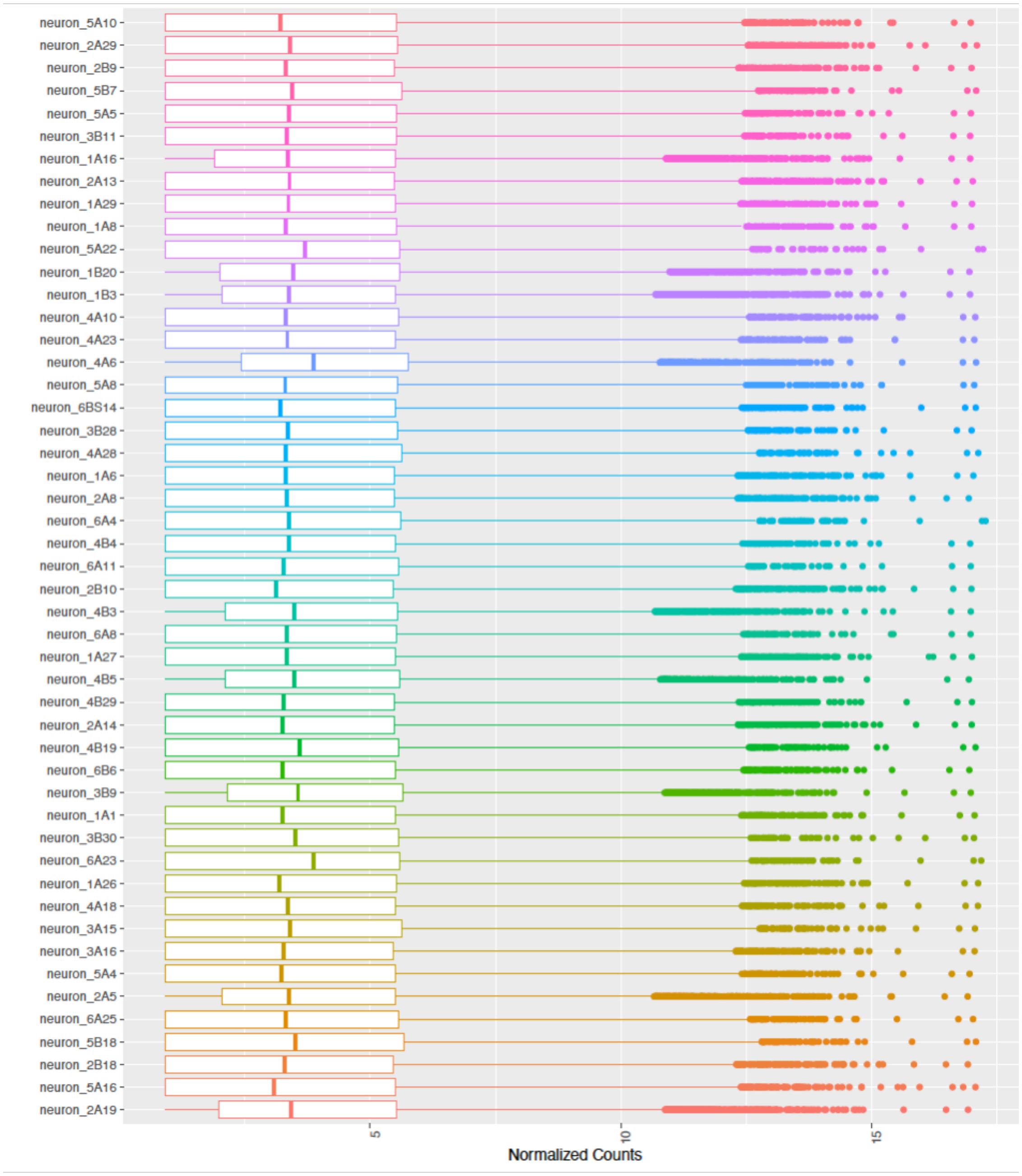

Supplementary Figure S2. Boxplots of Normalized Counts: This shows the expression profile of normalized read counts for each sample. The line dividing the box represents the median of the data and top and bottom of the box shows the upper and lower quartiles respectively. The whiskers show the highest and lowest values, excluding outliers, which are show as dots.

Supplementary Figure S3

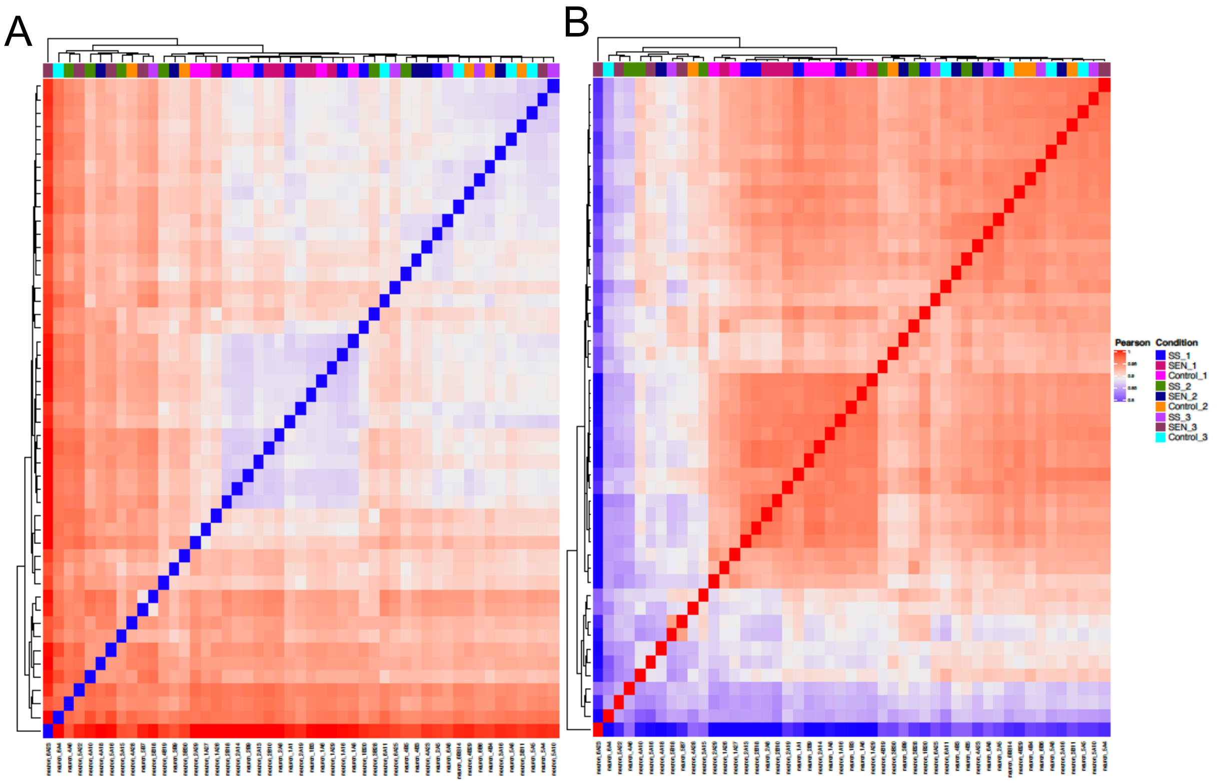

Supplementary Figure S3. Sample clustering by Euclidean distance (A) and Pearson Coefficient (B): This heatmap was computed by Euclidean distance of expression or Pearson Coefficient, based on normalized data. Color coding is by Euclidean distance and by group/condition. This figure can indicate the relationship between the samples and groups.

Supplementary Figure 4

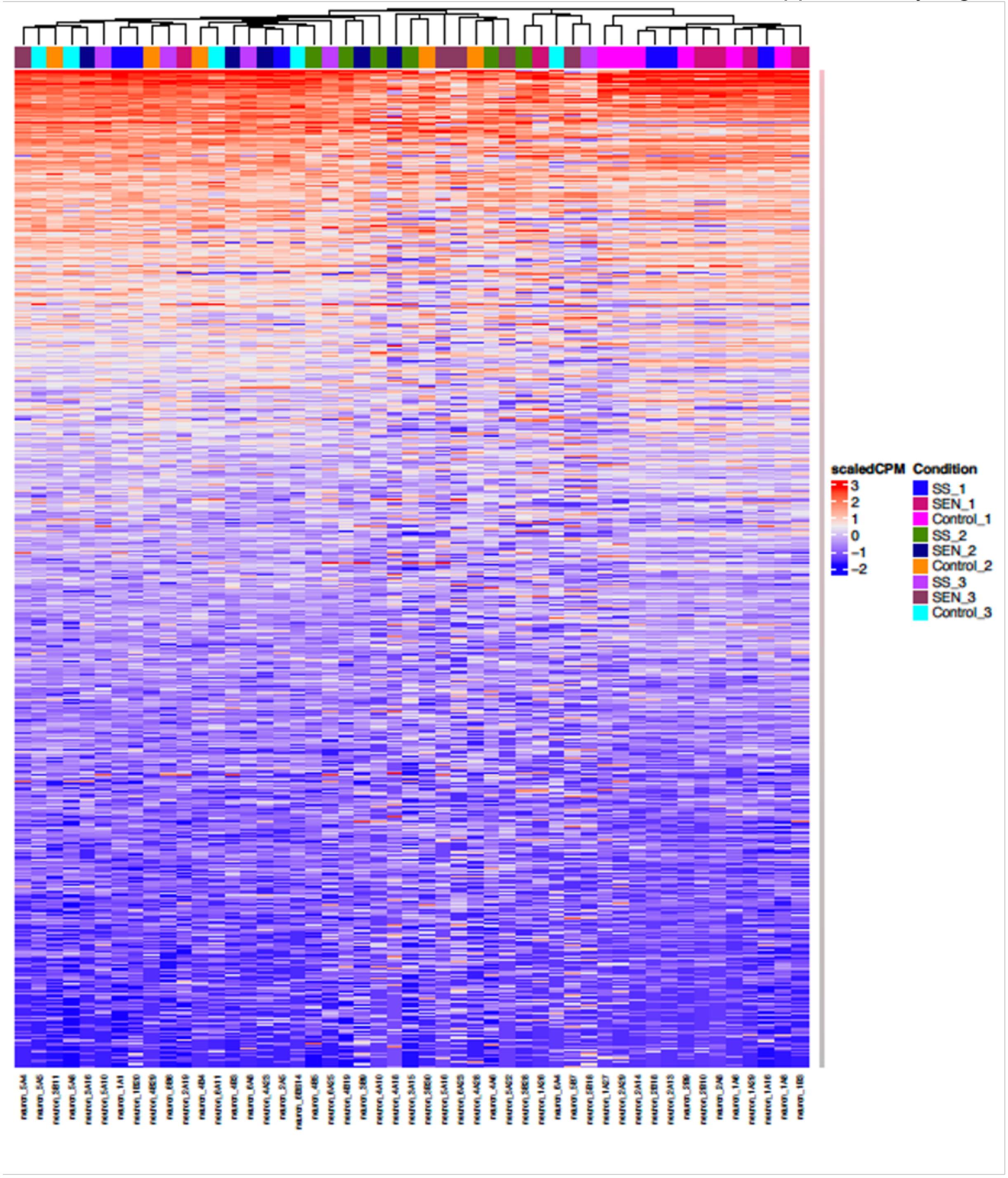

Supplementary Figure 4. Sample clustering based on CPM: The heatmap illustrates the sample correlation computed from gene expression count values. It shows how well the samples from each group cluster based on gene expression counts.

	Sample_ID	Total.Reads	Total.Mapped.Reads	Percent.Mapped.Reads	Percent.Properly.Paired	Uniquely.Mapped.Reads
1	neuron_1A1	25,147,209	13,716,647	54.55	N/A	7,868,457
2	neuron_1A16	24,614,204	15,018,586	61.02	N/A	9,719,071
3	neuron_1A26	15,956,737	11,178,014	70.05	N/A	5,570,566
4	neuron_1A27	17,381,408	11,404,020	65.61	N/A	7,353,832
5	neuron_1A29	19,462,373	11,933,519	61.32	N/A	7,411,854
6	neuron_1A6	16,997,262	10,767,027	63.35	N/A	6,647,216
7	neuron_1A8	19,990,820	12,384,512	61.95	N/A	7,896,098
8	neuron_1B20	21,545,235	11,214,580	52.05	N/A	7,258,734
9	neuron_1B3	18,196,888	10,716,868	58.89	N/A	6,994,667
10	neuron_2A13	16,790,726	9,618,303	57.28	N/A	6,081,449
11	neuron_2A14	20,143,794	12,369,836	61.41	N/A	7,937,518
12	neuron_2A19	20,030,849	11,425,780	57.04	N/A	7,710,816
13	neuron_2A29	16,286,305	10,333,423	63.45	N/A	5,736,895
14	neuron_2A5	19,592,710	9,099,347	46.44	N/A	6,067,134
15	neuron_2A8	18,747,882	11,194,319	59.71	N/A	7,700,894
16	neuron_2B10	20,446,081	12,667,391	61.96	N/A	8,336,729
17	neuron_2B18	17,667,409	10,186,141	57.65	N/A	7,167,543
18	neuron_2B9	18,775,904	11,412,693	60.78	N/A	7,381,753
19	neuron_3A15	20,024,858	5,017,164	25.05	N/A	2,815,103
20	neuron_3A16	13,774,167	7,815,738	56.74	N/A	4,551,463
21	neuron_3B11	18,249,746	9,386,404	51.43	N/A	5,913,496
22	neuron_3B28	13,000,625	6,881,402	52.93	N/A	4,127,176
23	neuron_3B30	13,957,806	6,915,372	49.54	N/A	4,046,256
24	neuron_3B9	22,150,477	8,129,814	36.70	N/A	4,949,299
25	neuron_4A10	15,387,350	5,812,581	37.78	N/A	3,278,309
26	neuron_4A18	16,365,586	8,525,768	52.10	N/A	4,124,382
27	neuron_4A23	13,015,260	6,794,951	52.21	N/A	3,881,710
28	neuron_4A28	14,689,784	4,826,424	32.86	N/A	2,384,614
29	neuron_4A6	21,302,505	6,789,358	31.87	N/A	3,413,520
30	neuron_4B19	11,937,374	6,677,110	55.93	N/A	3,613,661
31	neuron_4B29	12,630,104	6,726,679	53.26	N/A	4,178,482
32	neuron_4B3	17,375,788	8,444,054	48.60	N/A	4,998,329
33	neuron_4B4	17,412,315	8,727,570	50.12	N/A	5,433,350
34	neuron_4B5	19,078,835	7,665,855	40.18	N/A	4,752,644
35	neuron_5A10	36,465,847	18,173,442	49.84	N/A	11,406,386
36	neuron_5A16	13,572,744	5,691,586	41.93	N/A	3,356,602
37	neuron_5A22	9,581,067	5,783,121	60.36	N/A	2,421,279
38	neuron_5A4	19,050,978	9,304,611	48.84	N/A	6,057,198
39	neuron_5A5	21,611,023	10,524,443	48.70	N/A	6,515,426
40	neuron_5A8	13,987,900	7,226,102	51.66	N/A	4,186,984
41	neuron_5B18	12,716,601	8,258,939	64.95	N/A	4,678,873
42	neuron_5B7	13,749,928	7,383,040	53.70	N/A	4,166,817
43	neuron_6A11	22,375,910	9,884,760	44.18	N/A	5,959,494
44	neuron_6A23	15,797,836	1,744,182	11.04	N/A	752,520
45	neuron_6A25	13,773,811	6,330,866	45.96	N/A	3,680,929
46	neuron_6A4	10,507,069	7,122,174	67.78	N/A	2,698,354
47	neuron_6A8	25,894,054	10,271,603	39.67	N/A	6,188,794
48	neuron_6B6	17,047,885	8,872,134	52.04	N/A	5,808,803
49	neuron_6BS14	12,601,135	6,811,583	54.06	N/A	3,805,139

Supplementary Figure 5. Alignment Summary: Statistics for all samples listing proportions of reads mapped for each sample. Low percent mapped can indicate either poor read quality or contamination with nucleic acids of another organism.

Supplementary Figure 6

	Sample_ID	Percent.Exonic	Percent.Intronic	Percent.Intergenic	Total.Features	Features.With.Coverage	Avg.RPKM
1	neuron_1A1	100	0	0	38,711	25,547	5.690
2	neuron_1A16	100	0	0	38,711	26,970	5.721
3	neuron_1A26	100	0	0	38,711	24,131	5.919
4	neuron_1A27	100	0	0	38,711	25,285	5.625
5	neuron_1A29	100	0	0	38,711	25,985	5.630
6	neuron_1A6	100	0	0	38,711	25,190	5.647
7	neuron_1A8	100	0	0	38,711	25,624	5.775
8	neuron_1B20	100	0	0	38,711	27,184	5.688
9	neuron_1B3	100	0	0	38,711	26,218	5.845
10	neuron_2A13	100	0	0	38,711	25,654	5.805
11	neuron_2A14	100	0	0	38,711	25,279	5.768
12	neuron_2A19	100	0	0	38,711	26,810	5.923
13	neuron_2A29	100	0	0	38,711	25,629	5.646
14	neuron_2A5	100	0	0	38,711	26,234	5.719
15	neuron_2A8	100	0	0	38,711	25,967	5.912
16	neuron_2B10	100	0	0	38,711	24,643	5.766
17	neuron_2B18	100	0	0	38,711	25,917	6.025
18	neuron_2B9	100	0	0	38,711	25,303	5.902
19	neuron_3A15	100	0	0	38,711	24,229	5.532
20	neuron_3A16	100	0	0	38,711	24,089	5.342
21	neuron_3B11	100	0	0	38,711	25,724	5.606
22	neuron_3B28	100	0	0	38,711	24,912	5.703
23	neuron_3B30	100	0	0	38,711	24,822	5.198
24	neuron_3B9	100	0	0	38,711	26,796	5.439
25	neuron_4A10	100	0	0	38,711	23,554	5.369
26	neuron_4A18	100	0	0	38,711	24,803	5.281
27	neuron_4A23	100	0	0	38,711	24,899	5.243
28	neuron_4A28	100	0	0	38,711	23,292	5.231
29	neuron_4A6	100	0	0	38,711	26,908	5.450
30	neuron_4B19	100	0	0	38,711	25,851	5.329
31	neuron_4B29	100	0	0	38,711	24,386	5.599
32	neuron_4B3	100	0	0	38,711	26,669	5.584
33	neuron_4B4	100	0	0	38,711	25,325	5.647
34	neuron_4B5	100	0	0	38,711	26,437	5.621
35	neuron_5A10	100	0	0	38,711	25,772	5.686
36	neuron_5A16	100	0	0	38,711	22,952	5.093
37	neuron_5A22	100	0	0	38,711	22,949	4.642
38	neuron_5A4	100	0	0	38,711	24,623	5.765
39	neuron_5A5	100	0	0	38,711	26,121	5.589
40	neuron_5A8	100	0	0	38,711	24,357	5.222
41	neuron_5B18	100	0	0	38,711	24,903	5.353
42	neuron_5B7	100	0	0	38,711	24,381	5.337
43	neuron_6A11	100	0	0	38,711	25,440	5.575
44	neuron_6A23	100	0	0	38,711	22,080	4.949
45	neuron_6A25	100	0	0	38,711	25,092	5.341
46	neuron_6A4	100	0	0	38,711	22,361	4.836
47	neuron_6A8	100	0	0	38,711	26,100	5.488
48	neuron_6B6	100	0	0	38,711	25,235	5.853
49	neuron_6BS14	100	0	0	38,711	23,823	5.335

Supplementary Figure 6. Alignment to genomic component: Statistics for all samples listing the proportion of reads mapped to different genomic components, the number of genomic features with read coverage, and the average RPKM for each samples. High proportions of reads mapping to intergenic regions can indicate DNA contamination. Immature RNA contamination can cause a high proportion of the reads to map to intronic regions.

Supplementary Figure S7

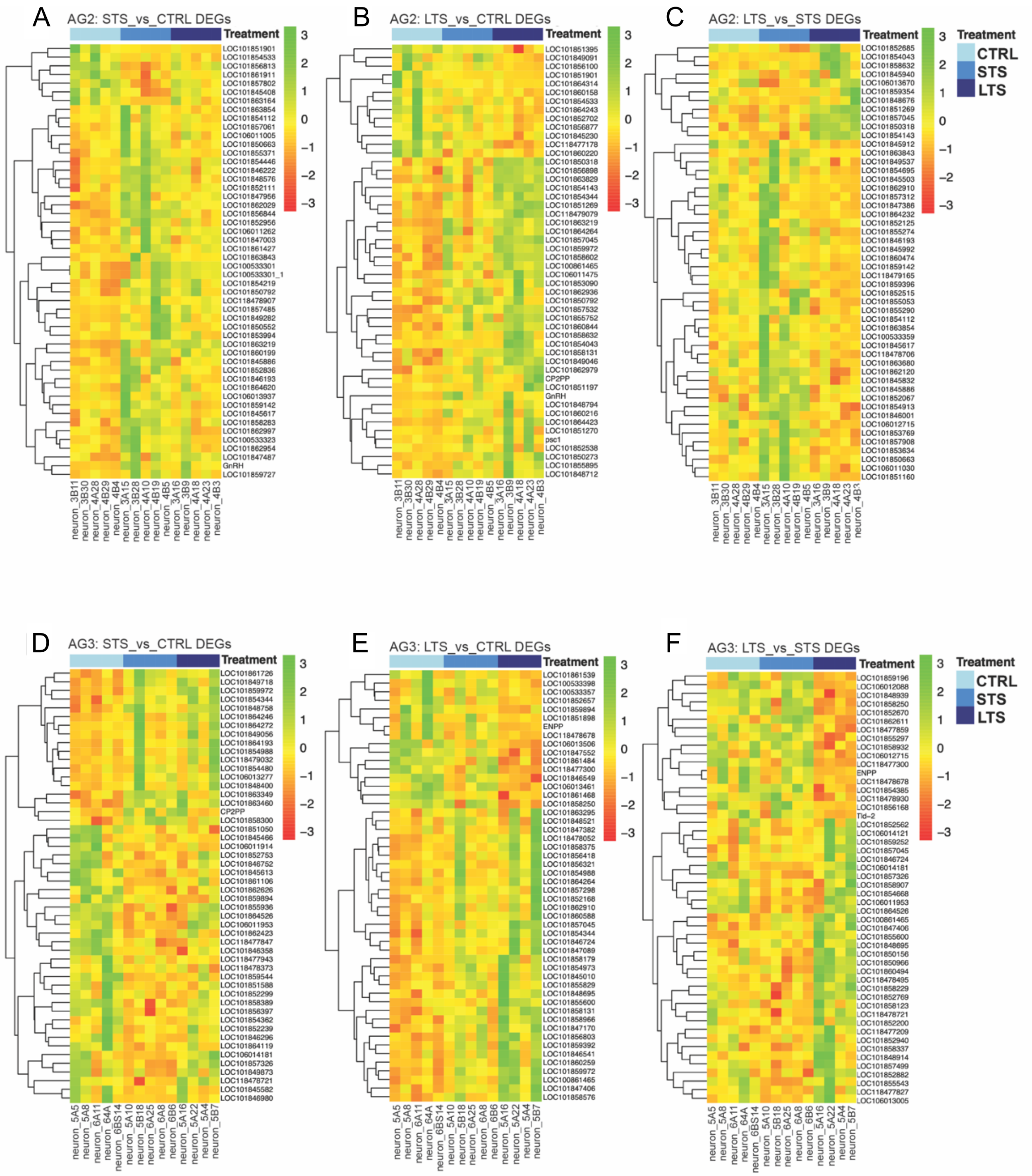

Supplementary Figure 7. Heat maps showing the normalized and scaled expression values of the top 50 differentially expressed genes when ranked by p-value. The color gradient from green to red represents high to low expression levels across the

samples. The genes are ordered by hierarchical clustering using Euclidean distance and complete clustering method while the samples are ordered by condition. **A–C.** Age 2 STS versus Control, Age 2 LTS versus Control, Age 2 LTS versus STS respectively. **D–F.** Age 3 STS versus Control, Age 3 LTS versus Control, Age 3 LTS versus STS, respectively (see Supplementary Table S3).

Supplementary Figure S8

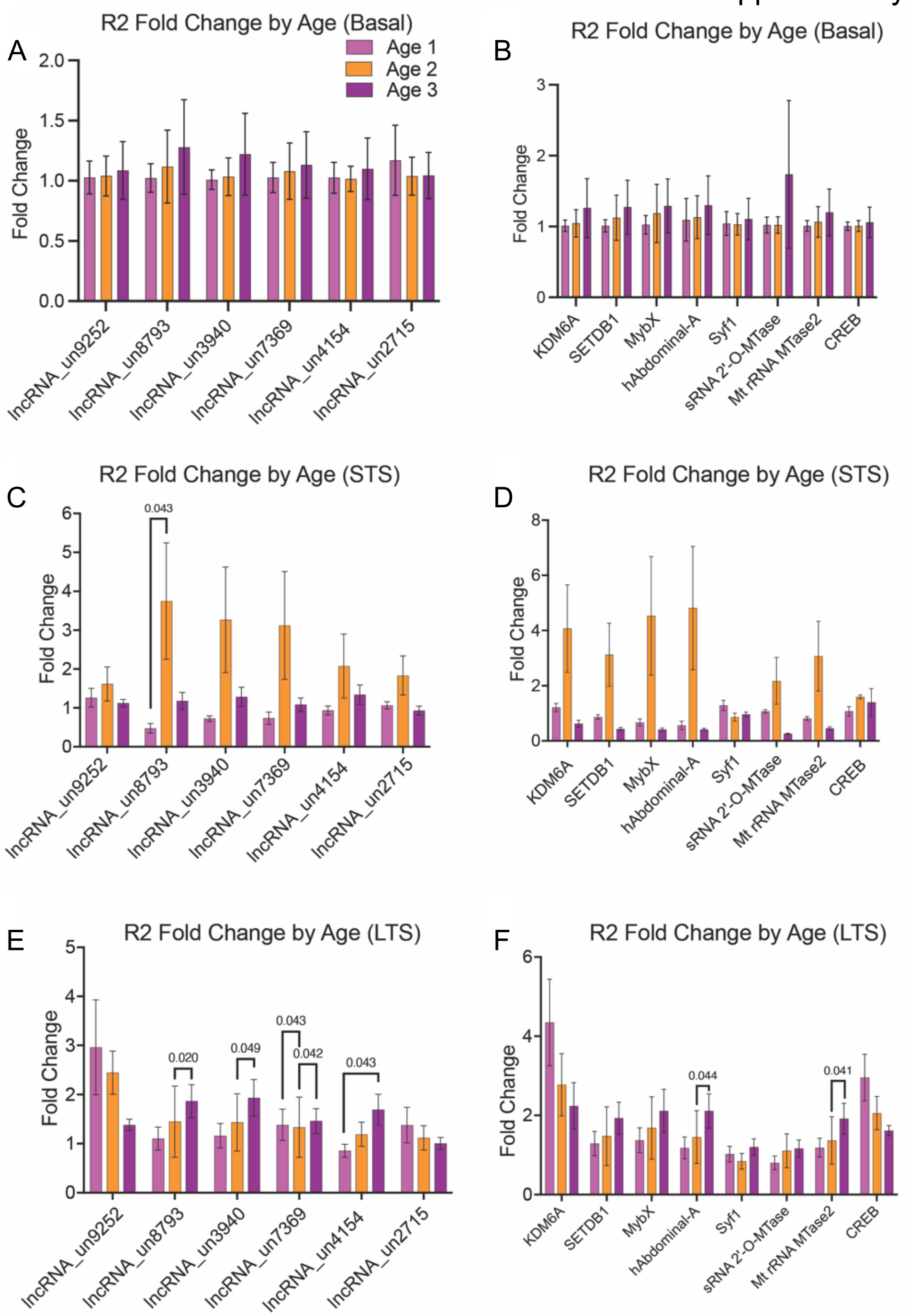

Supplementary Figure 8. qPCR analyses of RNAseq candidates in R2 across different age groups. This is a re-analysis of data from Figure 5. qPCR analyses of relative expression levels of lncRNAs and mRNAs in R2 : **A–B.** Basal condition, **C–D.** short-term sensitization, **E–F.** long-term sensitization. Relative gene expression levels are exhibited as the mean fold change, with error bars showing the SEM. One-way ANOVA followed by Tukey's post hoc test. N=5, p-Values are indicated in bar graphs (see Supplementary Table S5).

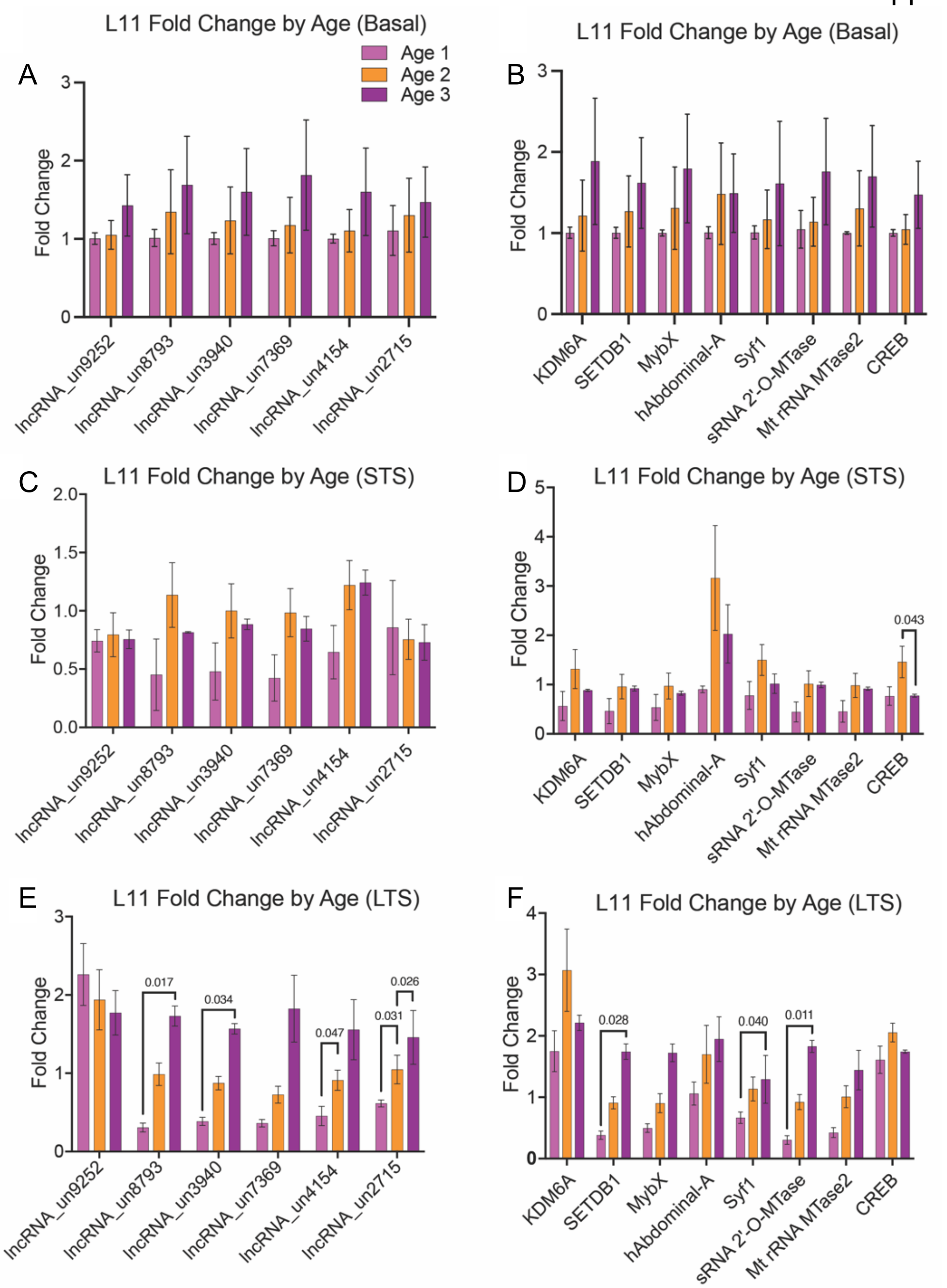

Supplementary Figure 9. qPCR analyses of RNAseq candidates in L11 across different age groups. This is a re-analysis of data from Figure 5. qPCR analyses of relative expression levels of lncRNAs and mRNAs in L11: **A–B.** Basal condition, **C–D.** short-term sensitization, **E–F.** long-term sensitization. Relative gene expression levels are exhibited as the mean fold change, with error bars showing the SEM. One-way ANOVA followed by Tukey's post hoc test. N=5, p-Values are indicated in bar graphs (see Supplementary Table S5).

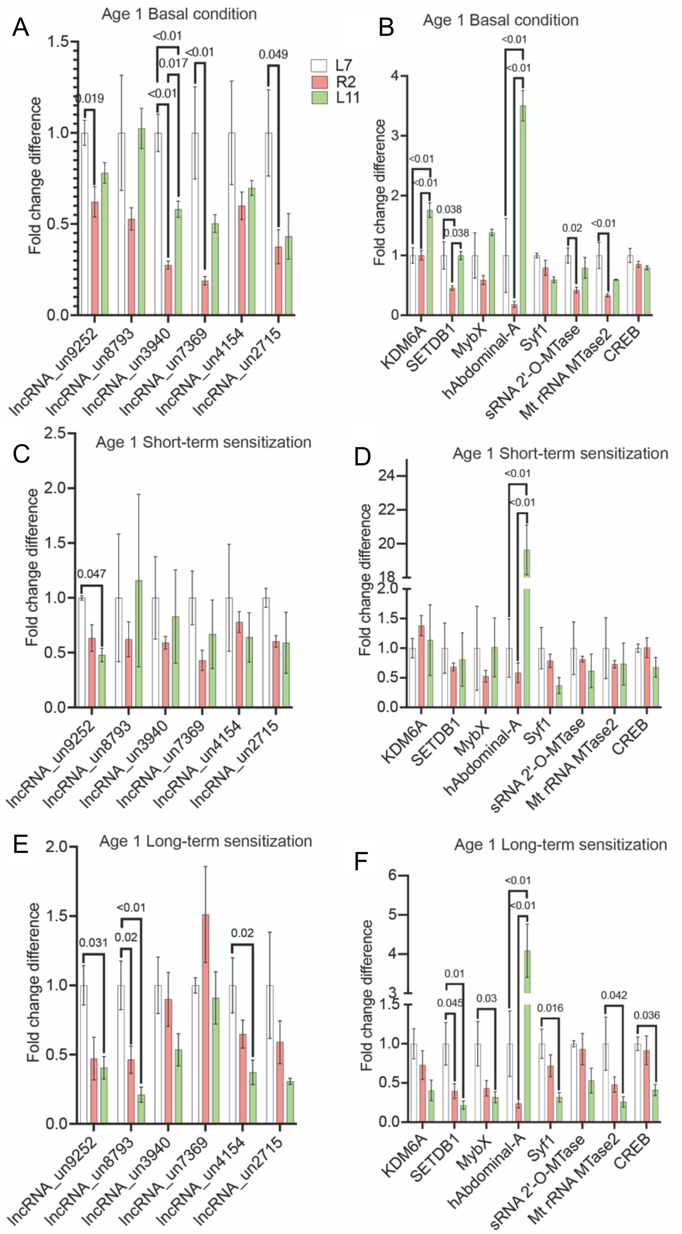

Supplementary Figure 10. Analysis of fold change of gene expression across L7, R2 and L11 in Age group 1. This is a re-analysis of data from Figures 1and 5. **A–B.** Basal condition, **C–D.** short-term sensitization, **E–F.** long-term sensitization. Relative gene expression levels are exhibited as the mean fold change, with error bars showing the SEM. One-way ANOVA followed by Tukey's post hoc test. N=5, p-Values are indicated in bar graphs (see Supplementary Table S5).

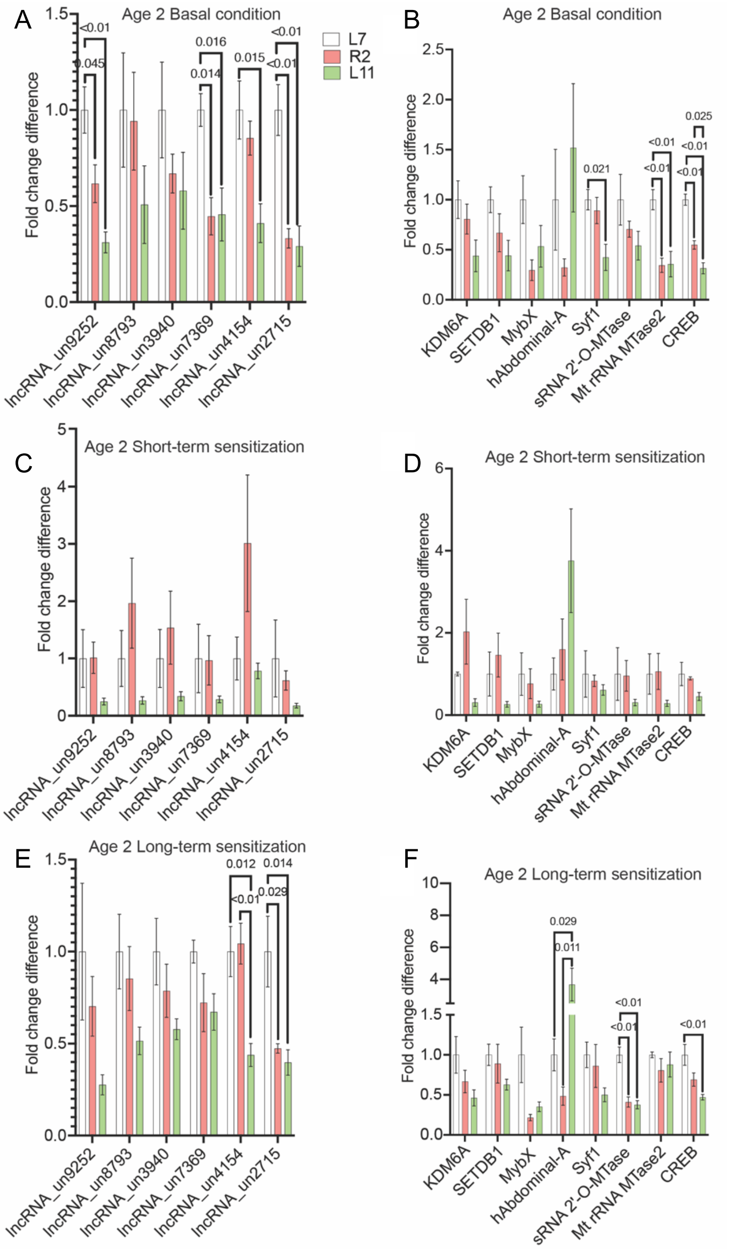

Supplementary Figure 11. Analysis of fold change of gene expression across L7, R2 and L11 in Age group 2. This is a re-analysis of data from Figures 3-5. **A–B.** Basal condition, **C–D.** short- term sensitization, **E–F.** long-term sensitization. Relative gene expression levels are exhibited as the mean fold change, with error bars showing the SEM. One-way ANOVA followed by Tukey's post hoc test. N=5, p-Values are indicated in bar graphs (see Supplementary Table S5).

Supplementary Figure S12

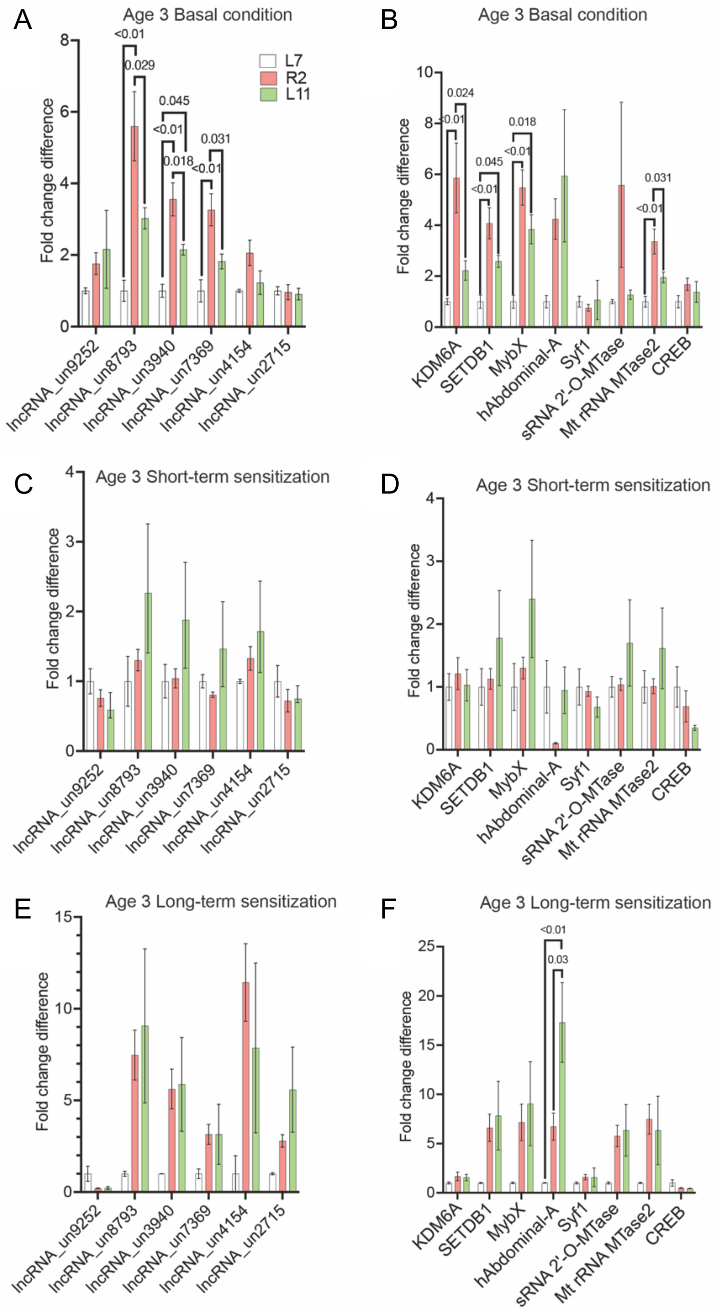

Supplementary Figure 12. Analysis of fold change of gene expression across L7, R2 and L11 in Age group 3. This is a re-analysis of data from Figures 3-5. **A–B.** Basal condition, **C–D.** short-term sensitization, **E–F.** long-term sensitization. Relative gene expression levels are exhibited as the mean fold change, with error bars showing the SEM. One-way ANOVA followed by Tukey's post hoc test. N=5, p-Values are indicated in bar graphs (see Supplementary Table S5).

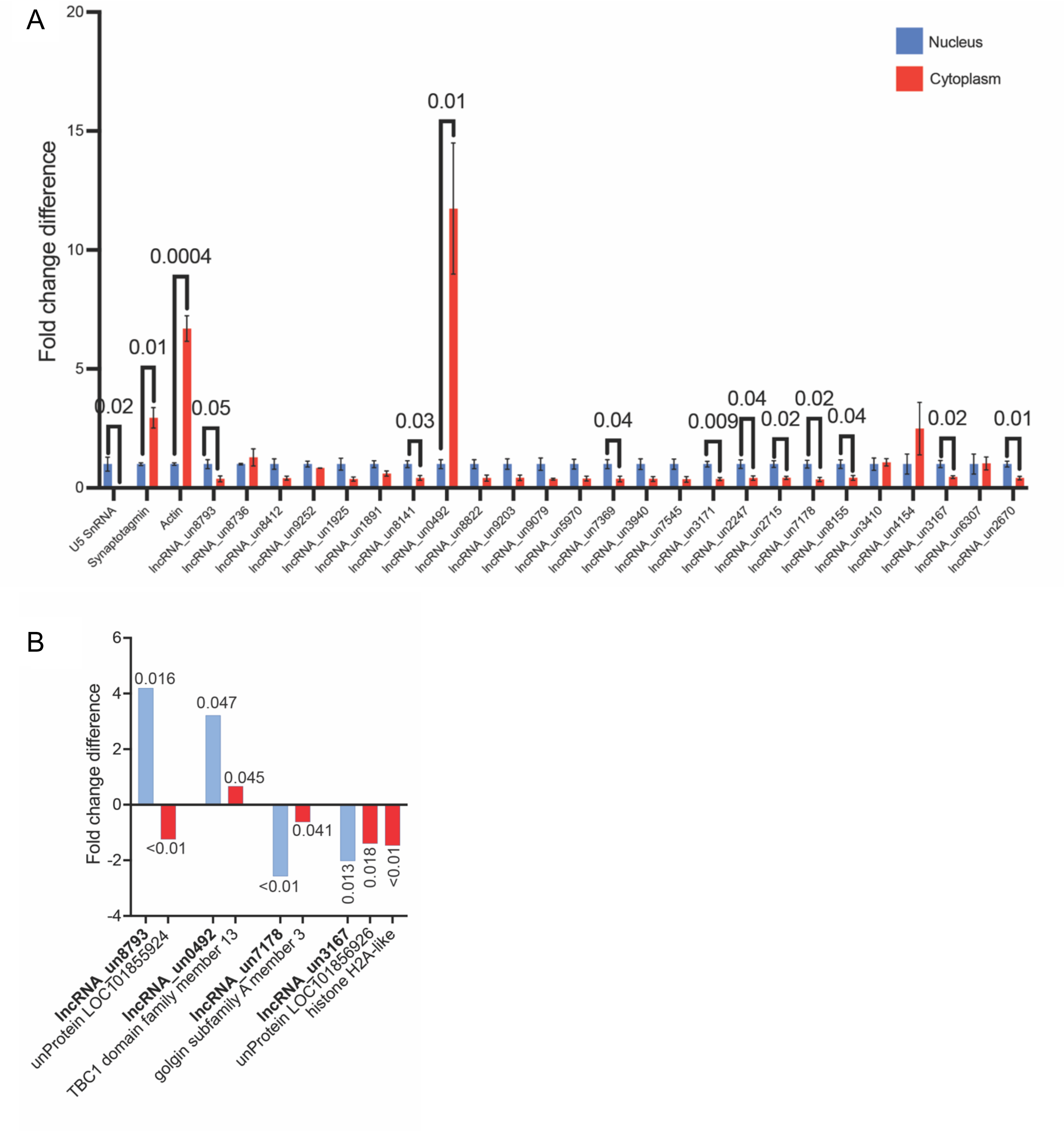

Supplementary Figure 13. Analyses of nuclear versus cytoplasmic localization of the lncRNAs. A. qPCR analyses of relative expression levels of 25 lncRNAs distribution. With error bars showing the SEM. Statistical analyses were conducted by paired two-tailed Student's T-test. **B.** Bioinformatic analyses based on NCBI and RNAseq data to identify lncRNA and cis-regulated transcripts. Relative gene expression levels shown as the mean fold change, p values are shown in bar graphs (see Supplementary Table S6).

Supplementary Tables

Supplementary table S1. Overview of single neuron analysis of aging associated changes in learning. Tables show Ages 1, 2, and 3 long-term sensitization (LTS) pre and post-test raw data, transformed data and 3-Way ANOVA analyses with multiple comparisons (related to Figure 1C), Age 1 and Age 3 (short-term sensitization) STS pre and post-test data and 1 hour post-test analyses (related to Supplementary figure S1), summary of single neuron Isolation, and summary of single neuron classification used for RNA seq analyses**.**

Supplementary table S2. RNAseq analysis of L7MN reveals specific changes in the expression of mRNAs and long-noncoding RNAs (lncRNAs) following STS and LTS training. Tables show age 1 upregulated mRNAs (related to figure 2A), age 1 downregulated mRNA (related to figure 2B), age 1 upregulated lncRNA (related to figure 2C), age 1 downregulated lncRNA (related to figure 2D), age 1 STS vs control downregulated lncRNAs, primer sequences (related to Figure 2), age 1 qPCR validation (Figure 2K and 2L).

Supplementary table S3. RNAseq analysis of L7MN from 10 and 12 months old Aplysia following STS and LTS training. Tables show Age 2 Upregulated RNAs (related to Figure 3A), Age 2 Down-regulated RNAs **(**related to Figure 3B), Age 2 Upregulated lncRNAs (related to Figure 3E), Age 2 Downregulated lncRNAs (related to Figure 3F), Age 3 Upregulated RNAs (related to Figure 3G), Age 3 Downregulated RNAs (related to Figure 3H), Age 3 Upregulated lncRNAs (related to Figure 3K), Age 3 Downregulated lncRNAs (related to Figure 3L), age 2 qPCR candidate validation (related to Figure 3M and 3N), AGE 3 qPCR candidate validation (related to Figure 3O and 2P).

Supplementary table S4. Analysis of aging associated changes in L7MN. Tables show total RNAs upregulated at basal conditions (related to Figure 4A), total RNAs downregulated at basal conditions (related to Figure 4B), total lncRNAs upregulated at basal conditions (related to Figure 4C), total lncRNAs downregulated at basal conditions (related to Figure 4D), comparison of L7 control qPCR candidates across ages, comparison of qPCR L7 STS candidates across ages, and comparison of qPCR L7 LTS candidates across ages.

Supplementary table S5. Gene expression analysis of R2 and L11 MN neurons following STS and LTS training. Tables show shows R2 age 1 qPCR candidate validation (related to Figure 5B and 5C), R2 age 2 qPCR candidate validation (related to Figure 5D and 5E), R2 age 3 qPCR candidate validation (related to Figure 5F and 5G), L11 age 1 qPCR candidate validation (related to Figure 5H and 5I), L11 age 3 qPCR candidate validation (related to Figure 5L and 5M), qPCR candidate validations: Age 1,2,3 Control, LTS and STS expression levels of qPCR candidates among L7, R2 and L11(related to Supplementary figures 9-13).

Supplementary table S6. Validation of RNA seq data by qPCR for lncRNAs and its cis pair (± 200kb). Tables show analyses of potential cis targets of lncRNAs, and lncRNA and their cis regulated transcripts (related to Figure 6 and Supplementary figure S6),
